# Distinct temporal trajectories and risk factors for Post-acute sequelae of SARS-CoV-2 infection

**DOI:** 10.3389/fmed.2023.1227883

**Published:** 2023-10-16

**Authors:** Chen Chen, Sairam Parthasarathy, Jacqueline M. Leung, Michelle J. Wu, Katherine A. Drake, Vanessa K. Ridaura, Howard C. Zisser, William A. Conrad, Victor F. Tapson, James N. Moy, Christopher R. deFilippi, Ivan O. Rosas, Bellur S. Prabhakar, Mujeeb Basit, Mirella Salvatore, Jerry A. Krishnan, Charles C. Kim

**Affiliations:** ^1^Verily Life Sciences, South San Francisco, CA, United States; ^2^Division of Pulmonary, Allergy, Critical Care and Sleep Medicine, University of Arizona, Tucson, AZ, United States; ^3^Providence Little Company of Mary Medical Center Torrance, Torrance, CA, United States; ^4^Department of Medicine, Cedars-Sinai Medical Center, Los Angeles, CA, United States; ^5^Department of Internal Medicine, Rush University Medical Center, Chicago, IL, United States; ^6^Inova Heart and Vascular Institute, Falls Church, VA, United States; ^7^Department of Medicine, Baylor College of Medicine, Houston, TX, United States; ^8^Department of Microbiology and Immunology, University of Illinois–College of Medicine, Chicago, IL, United States; ^9^Department of Internal Medicine, University of Texas Southwestern Medical Center, Dallas, TX, United States; ^10^Department of Medicine, Weill Cornell Medicine, New York, NY, United States; ^11^Department of Population Health Sciences, Weill Cornell Medicine, New York, NY, United States; ^12^Breathe Chicago Center, University of Illinois Chicago, Chicago, IL, United States

**Keywords:** PASC, symptom clusters, long COVID, SARS-CoV-2, COVID-19

## Abstract

**Background:**

The understanding of Post-acute sequelae of SARS-CoV-2 infection (PASC) can be improved by longitudinal assessment of symptoms encompassing the acute illness period. To gain insight into the various disease trajectories of PASC, we assessed symptom evolution and clinical factors associated with the development of PASC over 3 months, starting with the acute illness period.

**Methods:**

We conducted a prospective cohort study to identify parameters associated with PASC. We performed cluster and case control analyses of clinical data, including symptomatology collected over 3 months following infection.

**Results:**

We identified three phenotypic clusters associated with PASC that could be characterized as remittent, persistent, or incident based on the 3-month change in symptom number compared to study entry: remittent (median; min, max: −4; −17, 3), persistent (−2; −14, 7), or incident (4.5; −5, 17) (*p* = 0.041 remittent vs. persistent, *p* < 0.001 remittent vs. incident, *p* < 0.001 persistent vs. incident). Despite younger age and lower hospitalization rates, the incident phenotype had a greater number of symptoms (15; 8, 24) and a higher proportion of participants with PASC (63.2%) than the persistent (6; 2, 9 and 52.2%) or remittent clusters (1; 0, 6 and 18.7%). Systemic corticosteroid administration during acute infection was also associated with PASC at 3 months [OR (95% CI): 2.23 (1.14, 4.36)].

**Conclusion:**

An incident disease phenotype characterized by symptoms that were absent during acute illness and the observed association with high dose steroids during acute illness have potential critical implications for preventing PASC.

## Introduction

Severe acute respiratory syndrome coronavirus 2 (SARS-CoV-2) caused the coronavirus disease of 2019 (COVID-19) pandemic and can lead to new or persistent symptoms called Post-acute sequelae of SARS-CoV-2 (PASC), also known as “Long-COVID.” PASC has engendered another global health crisis, affecting tens of millions of people worldwide (1). PASC is defined as new or persistent symptoms that are present greater than 4 weeks after SARS-CoV-2 infection (2). In contrast, the World Health Organization (WHO) defined the “post-COVID-19 condition” as that which occurs in individuals with a history of probable or confirmed SARS-CoV-2 infection, usually 3 months from the onset of COVID-19, with symptoms that last for at least 2 months and cannot be explained by an alternative diagnosis. Despite the definitional variations, the economic costs of PASC are estimated at $2.6 trillion in the United States alone (2).

Differences in the virus variant and host response to the virus likely contribute to the risk, severity, and trajectory of PASC. The pathogenesis of PASC also likely varies and may include the failure to recover from severe microvascular injuries sustained during acute COVID-19, emergent autoimmune responses, viral persistence, gut dysbiosis, and dysregulated immune responses (3, 4). Such varied pathophysiology may lead to different temporal trends in the emergent or remitting nature of PASC that, besides creating measurement challenges, may also inform us of the underlying pathogenesis and treatment approaches. Despite such complexity, possibly involving different pathogenic mechanisms, most studies of PASC have taken a cross-sectional approach using concurrent controls with participants who test negative for COVID-19 but have COVID-like symptoms, historical controls, or uninfected controls (5). Moreover, at this time, a very limited number of longitudinal studies have assessed symptoms during both the acute illness and 3 months following SARS-CoV-2 illness (6, 7).

Our study objective was to explore the temporal pattern of COVID-19 symptoms by performing longitudinal cluster analysis of symptoms collected from participants enrolled in the Predictors of Severe COVID-19 Outcomes (PRESCO) study, who were enrolled as they presented for the management of acute COVID-19 during the first year of the COVID-19 pandemic and followed for 3 months. The secondary objective was to assess the parameters associated with PASC that also encompassed the acute illness period. Such a study performed during the early stages of the pandemic could yield a clearer picture of the pathogenesis of PASC without confounding from vaccines or antivirals.

## Materials and methods

### Study design

**FIGURE 1:**
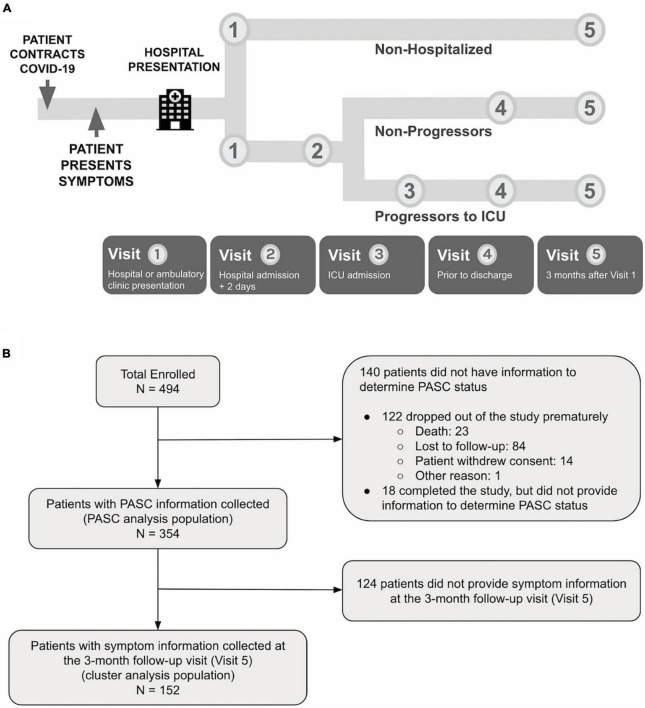
**(A)** PRESCO is a longitudinal COVID-19 study, enrolling participants across eight hospital sites in the United States. For each participant, clinical data is collected at up to five visits during SARS-CoV-2 infection and recovery, including at hospital or ambulatory clinic presentation, 2 days after hospital presentation, ICU admission, hospital discharge, and 3 months after hospital or clinic presentation. **(B)** A total of 494 participants were enrolled in the study, with 354 participants having PASC outcome information collected and 152 participants having 3-month follow-up (Visit 5) information on symptomatology collected.

The PRESCO study is a multi-center, prospective, 3-month cohort study designed to identify clinical and molecular signatures associated with progression to severe COVID-19. There were up to five study visits: (1) enrollment during initial presentation to a hospital or ambulatory clinic, and if occurred, (2) 2 days after hospitalization; (3) the day of intensive care unit (ICU) admission; (4) the day of hospital discharge; and (5) 3 months after enrollment (Figure 1A). Adults with laboratory-test confirmed SARS-CoV-2 infection (RT-PCR or antigen testing) who received care at eight sites (Table 1) between May 2020 and June 2021 were invited to participate. Eligible participants were adults that (1) were 18 years old or older in age, (2) were U.S. residents, (3) confirmed positive for COVID-19, (4) received care at a participating site, (5) were willing and able to provide informed consent, and (6) were willing and able to complete all study procedures. Participants were excluded if they were pregnant. Enrollment was completed before the delta variant became predominant in the United States in the summer of 2021 and before the availability of nirmatrelvir and ritonavir treatments. Later on in the study as the pandemic evolved, collection of PASC information and symptom information at the 3-month follow-up visit (Visit 5) were added. See the Supplementary material for more details.

**TABLE 1:** Demographics, patient characteristics, and disease characteristics are described for each analysis population, including the full enrolled population and population used for analysis.

Parameter	Enrolled population (*N* = 494)	Patients with PASC information collected (PASC analysis population) (*N* = 354)	Patients with symptoms collected at the 3-month follow-up (Cluster analysis population) (*N* = 152)
**Demographics**			
Age, *n*, mean (SD)	493, 50.5 (15.4)	354, 49.2 (15.1)	152, 46.8 (15.9)
**Sex**			
Female, *n* (%)	254 (51.5)	193 (54.5)	96 (63.2)
Male, *n* (%)	239 (48.5)	161 (45.5)	56 (36.8)
**Race**			
American Indian or Alaska Native, *n* (%)	2 (0.4)	1 (0.3)	1 (0.7)
Asian, *n* (%)	24 (4.9)	15 (4.2)	9 (5.9)
Black or African American, *n* (%)	152 (30.8)	113 (31.9)	42 (27.6)
Native Hawaiian or Other Pacific Islander, *n* (%)	1 (0.2)	0	0
White, *n* (%)	208 (42.1)	152 (42.9)	74 (48.7)
Other, *n* (%)	47 (9.5)	35 (9.9)	12 (7.9)
Unknown, *n* (%)	60 (12.1)	38 (10.7)	14 (9.2)
**Ethnicity**			
Hispanic, *n* (%)	171 (34.6)	124 (35.0)	43 (28.3)
Non-Hispanic, *n* (%)	297 (60.1)	217 (61.3)	104 (68.4)
Unknown, *n* (%)	26 (5.3)	13 (3.7)	5 (3.3)
Race - Ethnic Groups			
Asian, *n* (%)	24 (4.9)	15 (4.2)	9 (5.9)
Black or African American, *n* (%)	152 (30.8)	113 (31.9)	42 (27.6)
Non-Hispanic White, *n* (%)	111 (22.5)	80 (22.6)	45 (29.6)
Other, *n* (%)	196 (39.7)	140 (39.5)	51 (33.6)
Unknown, *n* (%)	11 (2.2)	6 (1.7)	5 (3.3)
**Site**			
Baylor College of Medicine, *n* (%)	81 (16.4)	65 (18.4)	36 (23.7)
Cedars-Sinai Medical Center, *n* (%)	15 (3.0)	10 (2.8)	9 (5.9)
Cornell, *n* (%)	41 (8.3)	11 (3.1)	2 (1.3)
Inova Health Care Services, *n* (%)	101 (20.4)	74 (20.9)	29 (19.1)
Rush University Medical Center, *n* (%)	89 (18.0)	75 (21.2)	30 (19.7)
UT Southwestern (UTSW), *n* (%)	23 (4.7)	12 (3.4)	3 (2.0)
University of Arizona, *n* (%)	18 (3.6)	10 (2.8)	10 (6.6)
University of Illinois, Chicago, *n* (%)	126 (25.5)	97 (27.4)	33 (21.7)
**BMI group**			
< 30 kg/m^2^, *n* (%)	220 (44.7)	161 (45.5)	71 (46.7)
≥ 30 kg/m^2^, *n* (%)	255 (51.8)	181 (51.1)	73 (48.0)
Unknown, (%)	17 (3.5)	12 (3.4)	8 (5.3)
**Tobacco use**			
No, *n* (%)	361 (73.5)	257 (72.8)	113 (74.3)
Yes, *n* (%)	130 (26.5)	96 (27.2)	39 (25.7)
**Comorbidities, *n* (%)**			
Atrial fibrillation	16 (3.2)	16 (4.5)	5 (3.3)
Hypertension	163 (33.0)	163 (46.0)	60 (39.5)
Coronary artery disease	16 (3.2)	16 (4.5)	8 (5.3)
Hyperlipidemia	57 (11.5)	57 (16.1)	18 (11.8)
Anemia	19 (3.8)	19 (5.4)	10 (6.6)
Gastroesophageal reflux disease	21 (4.3)	21 (5.9)	13 (8.6)
Asthma	43 (8.7)	43 (12.1)	16 (10.5)
Chronic kidney disease	23 (4.7)	23 (6.5)	5 (3.3)
Type 2 diabetes mellitus	87 (17.6)	87 (24.6)	26 (17.1)
**Disease progression**			
WHO score, *n*, median (min, max)	494, 4.0 (2.0, 8.0)	354, 4.0 (2.0, 7.0)	152, 3.0 (2.0, 5.0)
Days from COVID-19 start to hospital admission, *n*, mean (SD)	399, 3.0 (4.7)	274, 3.0 (4.0)	94, 2.5 (2.2)
Hospital duration in days, *n*, mean (SD)	390, 6.9 (6.8)	274, 6.1 (5.7)	94, 5.2 (3.7)
**Cohort**			
Ambulatory, *n* (%)	95 (19.2)	80 (22.6)	58 (38.2)
Hospitalized, *n* (%)	399 (80.8)	274 (77.4)	94 (61.8)
Intubated, *n* (%)	15 (3.0)	4 (1.1)	0
Admitted to ICU, *n* (%)	16 (3.2)	4 (1.1)	0
Death, *n* (%)	23 (4.7)	0	0

The PRESCO study was approved by a central Western Institutional Review Board (Protocol# 20201016) and at each of the eight sites. Written informed consent was obtained from all participants or their legally authorized representatives before study-related procedures were performed.

### Measures

Participants were asked to select symptoms present from a list of 22 symptoms at enrollment and a list of 30 at the 3-month follow-up (Supplementary Tables 1, 2). Symptoms were grouped and analyzed by System Organ Class (SOC) according to the Medical Dictionary for Regulatory Activities (MedDRA), which groups symptoms by etiology, manifestation site, and/or purpose. At the 3-month follow-up visit, participants were also asked how many weeks had passed since their last study visit until they felt at their usual state of health. We defined PASC as those individuals who did not recover to their usual state of health for four or more weeks after the start of COVID-19, which was determined by the earliest of several non-self-reported dates, including enrollment, first positive SARS-CoV-2 test, hospital presentation, and hospitalization. WHO clinical severity scale was used to measure COVID-19 severity (8). Additionally, participants’ demographics and longitudinal clinical characteristics were collected. See the Supplementary material for more details.

### Statistical analysis

The nomenclatures for the populations used in the analysis are provided in Figure 1B. The enrolled population included participants who signed the informed consent and were enrolled in the study. The PASC analysis population included those who had sufficient data to be categorized as having PASC or without PASC (non-PASC), and the cluster analysis population included those who provided symptom information at the 3-month follow-up visit. The PASC analysis population included 354 participants out of the 494 (71.7%) participants enrolled. Descriptive statistics included mean (standard deviation, SD) or median (range), and frequencies (percentages), as appropriate. Continuous data were compared using Wilcoxon rank sum tests, and categorical data were compared using Chi-square or Fisher’s exact tests, as appropriate. Unadjusted univariate tests were conducted for all demographic information, clinical characteristics, and clinical labs to search for statistically significant differences between PASC and non-PASC groups. Association analysis of PASC with comorbidities, concomitant medication, and clinical labs were further adjusted for potential confounders. Multiplicity was corrected in the association analysis of PASC with comorbidities, concomitant medications, and clinical labs, controlling the false discovery rate (FDR) at 0.05 with the Benjamini-Hochberg procedure. The cluster analysis population included 152 participants out of the 494 (30.8%) enrolled. Clustering analysis was based on symptoms collected in the questionnaire at the 3-month follow-up visit. Hierarchical clustering of participants was performed with Ward’s method using hamming distance. Three clusters were determined from visual evaluation of the heatmap, and the dendrogram was then cut at an appropriate height to generate resulting clusters. See the Supplementary material for more details.

Due to delayed implementation of the amendment to outcome survey, PASC information and 3-month follow-up symptoms were not collected from participants who exited the study before May 2021. Missing data was not imputed given the observational nature of the study.

## Results

A total of 494 participants were enrolled in the PRESCO study (Supplementary Figure 1). Demographics of the 494 enrolled participants revealed that most patients were Non-Hispanic White patients or African American patients (Table 1). Hypertension was the most common comorbidity at presentation, followed by type 2 diabetes mellitus, hyperlipidemia, and asthma (Table 1) with a median WHO severity score of 4 (range: 2 to 8; Table 1).

Of the 494 participants, 354 (71.6%) participants had Visit 5 information that could be used for studying PASC (termed the “*PASC analysis population*”; Figure 1B). Among the PASC analysis population, 137 (38.7%) participants were categorized as having PASC, and the remaining 217 (61.3%) participants were defined as without PASC.

### PASC associations

Based on the PASC analysis population, we analyzed the clinical characteristics that were associated with the development of PASC. Participants who developed PASC were significantly older than participants without PASC (*p* < 0.001; Table 2), but there were no sex differences. The PASC group had a greater proportion of Non-Hispanic White people and a lower proportion of Asians, Black people, and Hispanic people when compared to the non-PASC group (*p* < 0.01). Among comorbidities, there was a greater proportion of obesity and tobacco use in the PASC group compared to the non-PASC group (*p* = 0.007 and *p* = 0.04, respectively; Table 2). Hypertension and gastroesophageal reflux disease (GERD) were associated with PASC (Table 2 and Supplementary Table 3). Individuals who developed PASC had more severe COVID-19 than participants without PASC (*p* < 0.001; Table 2). The PASC group also had a higher proportion of patients that were hospitalized (*p* = 0.02; Table 2) and required a longer duration of hospitalization (*p* < 0.001; Table 2).

**TABLE 2:** Demographics, patient characteristics, and disease characteristics are described for PASC and non-PASC populations, including statistical comparisons between the two groups.

	PASC (*N* = 137)	Non-PASC (*N* = 217)	Effect size	*P*-value	FDR corrected *p*-value	Odds ratio (95% CI) adjusted for covariates
**Demographics**						
Age, *n*, mean (SD)	137, 52.7 (13.8)	217, 47.0 (15.4)	0.3900	0.0006		
**Sex, *n* (%)**						
Female	76 (55.5)	117 (53.9)	0.0313	0.8595		
Male	61 (44.5)	100 (46.1)				
**Race, *n* (%)**						
American Indian or Alaska Native	0 (0.0)	1 (0.5)		0.6595		
Asian	5 (3.6)	10 (4.6)				
Black or African American	39 (28.5)	74 (34.1)				
White	64 (46.7)	88 (40.6)				
Other	14 (10.2)	21 (9.7)				
Unknown	15 (10.9)	23 (10.6)				
**Ethnicity, *n* (%)**						
Hispanic	39 (28.5)	85 (39.2)	−0.2269	0.0855		
Non-Hispanic	90 (65.7)	127 (58.5)				
Unknown	8 (5.8)	5 (2.3)				
**Race - Ethnic Groups, *n* (%)**						
Asian	5 (3.6)	10 (4.6)		0.0022		
Black or African American	39 (28.5)	74 (34.1)				
Non-Hispanic White	45 (32.8)	35 (16.1)				
Other	44 (32.1)	96 (44.2)				
Unknown	4 (2.9)	2 (0.9)				
**Site, *n* (%)**						
Baylor College of Medicine	25 (18.2)	40 (18.4)		0.0034		
Cedars-Sinai Medical Center	3 (2.2)	7 (3.2)				
Weill Cornell Medicine	8 (5.8)	3 (1.4)				
Inova Health Care Services	19 (13.9)	55 (25.3)				
Rush University Medical Center	31 (22.6)	44 (20.3)				
UT Southwestern (UTSW)	10 (7.3)	2 (0.9)				
University of Arizona	4 (2.9)	6 (2.8)				
University of Illinois, Chicago	37 (27.0)	60 (27.6)				
**BMI group, *n* (%)**						
< 30 kg/m^2^	52 (38.0)	109 (50.2)	−0.2479	0.0143		
≥ 30 kg/m^2^	83 (60.6)	98 (45.2)				
Unknown	2 (1.5)	10 (4.6)				
**Tobacco use, *n* (%)**						
No	91 (66.4)	167 (77.0)	−0.2348	0.0405		
Yes	46 (33.6)	50 (23.0)				
**Common patient-reported comorbidities, *n* (%)**						
Anemia	9 (6.6)	10 (4.6)	0.0860	0.4719	0.5243	
Asthma	20 (14.6)	23 (10.6)	0.1210	0.3163	0.5243	
Atrial fibrillation	10 (7.3)	6 (2.8)	0.2130	0.0640	0.2132	
Chronic kidney disease	12 (8.8)	11 (5.1)	0.1470	0.1883	0.3765	
Coronary artery disease	8 (5.8)	8 (3.7)	0.1020	0.4320	0.5243	
Gastroesophageal reflux disease	15 (10.9)	6 (2.8)	0.3400	0.0022	0.0153	3.85 (1.31, 11.36)*
Hyperlipidemia	27 (19.7)	30 (13.8)	0.1580	0.1811	0.3765	
Hypertension	77 (56.2)	86 (39.6)	0.3330	0.0031	0.0153	1.38 (0.80, 2.37)
Type 2 Diabetes mellitus	35 (25.5)	52 (24.0)	0.0370	0.8001	0.8001	
**Disease progression**						
WHO score, *n*, median (min, max)	137, 4.0 (2.0, 7.0)	217, 3.0 (2.0, 7.0)	0.39	0.0004		
Days from COVID-19 start to hospital admission, *n*, mean (SD)	115, 3.8 (5.3)	159, 2.4 (2.5)	0.36	0.0786		
Hospital duration in days, *n*, mean (SD)	115, 7.8 (7.6)	159, 4.9 (3.3)	0.53	< 0.0001		
Cohort, *n* (%)						
Ambulatory care	22 (16.1)	58 (26.7)	−0.262	0.0273		
Hospitalized	115 (83.9)	159 (73.3)				
**Concomitant medication, *n* (%)**						
Acetaminophen	98 (71.5)	134 (61.8)	0.208	0.0665	0.1497	
Azithromycin	25 (18.2)	35 (16.1)	0.056	0.6632	0.8526	
Ceftriaxone	13 (9.5)	19 (8.8)	0.025	0.8503	0.9565	
Dexamethasone	90 (65.7)	87 (40.1)	0.519	<0.0001	<0.0001*	2.23 (1.14, 4.36)*
Enoxaparin	81 (59.1)	105 (48.4)	0.216	0.0503	0.1497	
Furosemide	12 (8.8)	24 (11.1)	−0.077	0.5891	0.8526	
Heparin	13 (9.5)	16 (7.4)	0.076	0.5517	0.8526	
Ibuprofen	16 (11.7)	25 (11.5)	0.005	>0.9999	>0.9999	
Remdesivir	69 (50.4)	68 (31.3)	0.39	0.0005	0.0022*	1.78 (0.97, 3.27)
**Clinical labs, *n*, median (min, max)**						
CBC - Absolute Lymphocyte Count, 10^3^ cells/dL (Visit 1)	118, 0.895 (0.15, 4.51)	167, 1.29 (0.18, 89.0)	−0.153	0.0001	0.0047	
Renal Function Test–Carbon Dioxide, Total mmol/L (Visit 4)	69, 25.0 (8.7, 35.0)	71, 23.0 (17.0, 33.0)	0.476	0.0013	0.0458	

Values in columns 4 to 6 indicate results from our unadjusted univariate analysis, and values in column 7 indicate results from our adjusted analysis.

* Indicates that the concomitant medication stays significant after propensity score analysis to account for treatment assignment bias. FDR corrected *p*-value was obtained using the Benjamini-Hochberg procedure to control the false discovery rate at 5%.

Dexamethasone and remdesivir usage were significantly greater in the PASC group compared to the non-PASC group (*p* < 0.001 and *p* = 0.002, respectively; Table 2) (9, 10). After adjusting for propensity score and final COVID-19 severity, the odds ratio (95% confidence interval [CI]) of developing PASC with treatment when compared to without treatment was 2.23 (95% CI; 1.14, 4.36) and 1.78 (95% CI; 0.97, 3.27) for dexamethasone and remdesivir, respectively.

### Laboratory abnormalities

Participants who developed PASC had significantly lower absolute lymphocyte counts at Visit 1, during the acute illness period (*p* = 0.005; Table 2). Although this was no longer statistically significant after adjusting for age and baseline COVID-19 severity (Supplementary Table 4), the effect size is large (effect size = −0.932). We also found significantly higher serum bicarbonate at the time of hospital discharge in the PASC group than the non-PASC group (*p* = 0.05; Supplementary Table 4). However, the difference was no longer significant after adjustment for confounders.

Multivariable modeling was conducted using clinical risk factors that were found significant in the univariate analysis (Supplementary Table 5). Dexamethasone administration, hospital duration, WHO score, lymphocyte count at hospital presentation, serum bicarbonate levels at hospital discharge, and body mass index were associated with PASC (Supplementary Table 5). Sensitivity analysis was performed excluding serum bicarbonate levels (available in only 122 patients) and revealed that in addition to the above PASC associations, GERD, tobacco use, and race-ethnicity were found to be associated with PASC, whereas BMI was excluded from the final model (Supplementary Table 5).

### Identification of clusters

A subset of 152 (42.9%) participants of the 354 PASC analysis population provided the type of symptoms they experienced during the 3-month follow-up period, which was used to perform cluster analyses (termed the “*cluster analysis population*”; Figure 1B). The cluster analysis population was slightly younger and had a greater proportion of women, a lower median WHO score, and a shorter time-period between COVID-19 onset and presentation to a health system compared to the enrolled population (Table 1).

**FIGURE 2:**
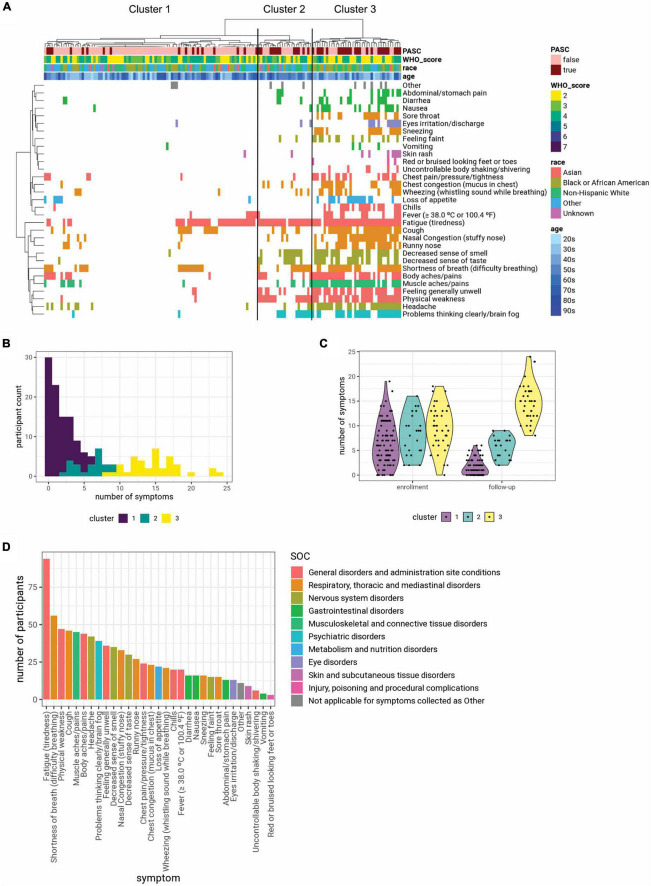
**(A)** Clustering participants based on symptoms collected at the 3-month follow-up visit (Visit 5) yielded three distinct clusters. Symptoms are colored based on system organ class (SOC), using the color scheme shown in panel **(D)**. The symptom clusters (1, 2, and 3) are shown in ascending order of average number of symptoms. **(B)** The histogram shows the distribution of the number of symptoms reported by each participant at the 3-month follow-up visit (Visit 5), colored by cluster identity. **(C)** While the three clusters have similar numbers of symptoms at enrollment, cluster 1 has fewer symptoms at the 3-month follow-up visit, while cluster 3 has more symptoms. **(D)** Summary of symptoms collected at the 3-month follow-up visit and their SOC.

Hierarchical clustering of the presence or absence of long-term symptoms revealed three distinct clusters of individuals (cluster 1: remitting, cluster 2: persistent, cluster 3: incident) (Figure 2A). All three clusters had similar symptom burden during the acute illness, but differed with regards to symptom burden at the 3-month follow-up visit, underscoring three different disease trajectories of COVID-19 when assessing temporal trends (Figures 2B, C). Participants in cluster 2 were on average older than those in cluster 3 (*p* = 0.015). There was a greater proportion of Asians in cluster 1 than in the other two clusters (Supplementary Table 6). There were no differences in sex, obesity, other comorbidities, hospitalization rate, or concomitant medication across the clusters. Interestingly, there was no significant difference in COVID severity (WHO ordinal scores) across clusters. Yet, cluster 1 had significantly lower rates of PASC compared to the other two clusters and a significant reduction in symptom number from the acute illness period, suggesting a “remitting” temporal phenotype (Figure 2C and Table 3). In contrast, cluster 2 demonstrated persistent symptomatology at 3-months compared to the acute illness period, suggesting a “persistent” temporal phenotype (Figure 2C and Table 3), and cluster 3 showed an increase in symptoms at 3 months that would suggest an “incident” temporal phenotype despite lower hospitalization rates (Figure 2C and Table 3). A preponderance of symptoms involving the neurological, respiratory, and general symptoms distinguished cluster 3 from cluster 1 (Supplementary Figure 2). While the number of days from the start of COVID-19 to Visit 5 was significantly greater in cluster 1 than cluster 3 (145.6 days in cluster 1 vs. 112.3 days in cluster 3; Table 3), cluster 1 still had a significantly larger symptom reduction compared to cluster 3 following regression analysis adjusting for that time influence (Supplementary Table 7). Multiple differences in clinical laboratory results at enrollment and at 3 months across clusters were also observed (Supplementary Table 8).

**TABLE 3:** Disease characteristics are described for each of the symptom clusters.

	Cluster 1 (*N* = 91)	Cluster 2 (*N* = 23)	Cluster 3 (*N* = 38)
Count of symptoms at study entry, *n*, median (min, max)	91, 6.0 (0.0, 19.0)	23, 9.0 (2.0, 16.0)	38, 10.0 (0.0, 18.0)$
Count of symptoms at the 3-month follow-up visit (Visit 5), *n*, median (min, max)	91, 1.0 (0.0, 6.0)	23, 6.0 (2.0, 9.0)*#	38, 15.0 (8.0, 24.0)$
Change in symptom counts from study entry to the 3-month follow-up visit (Visit 5), *n*, median (min, max)	91, −4.0 (−17.0, 3.0)	23, −2.0 (−14.0, 7.0)*#	38, 4.5 (−5.0, 17.0)$
WHO score, *n*, median (min, max)	91, 3.0 (2.0, 4.0)	23, 3.0 (2.0, 4.0)	38, 2.0 (2.0, 5.0)
Proportion of participants with PASC, *n* (%)	17 (18.7)	12 (52.2)*	24 (63.2)$
Hospitalized, *n* (%)	61 (67.0)	15 (65.2)	18 (47.4)
Hospitalized who developed PASC, *n* (%)	13 (14.3)	7 (30.4)	15 (39.5)$
Hospital duration (days), *n*, mean (SD)	61, 5.0 (3.3)	15, 5.3 (2.7)	18, 6.1 (5.4)
Days from COVID-19 start to hospital admission, *n*, mean (SD)	61, 2.6 (2.4)	15, 2.3 (2.1)	18, 1.9 (1.5)
Days from COVID-19 start to the 3-month follow-up visit (Visit 5), *n*, mean (SD)	91, 145.6 (73.5)	23, 119.2 (32.6)	38, 112.3 (38.6)$

Statistical significance between each pair of clusters is labeled using * to indicate statistical significant difference between Cluster 1 and 2, $ for Cluster 1 and 3, and # for Cluster 2 and 3. Follow-up visit (Visit 5) is the visit when the outcome survey was collected, in which participants reported symptoms that they experienced since their previous visit.

To further characterize these phenotypic clusters, we compared the type of symptoms present at 3 months classified by SOC. Constitutional symptoms included under “*General disorders and administration site conditions*” were the most frequent symptoms reported by patients in all three clusters at the 3-month follow-up visit, although their prevalence was significantly higher in clusters 2 and 3 compared to cluster 1 (Figure 2D and Supplementary Tables 2, 9). The majority of individuals in cluster 3 experienced symptoms relating to the *Nervous system*, and *Respiratory, thoracic and mediastinal disorders*. Cluster 3 participants also had a significantly higher prevalence of symptoms relating to *Gastrointestinal disorders* compared to the other two clusters (Figure 2D and Supplementary Tables 2, 9).

To better characterize the evolution of symptoms, we analyzed the longitudinal changes in symptomatology by SOC for each cluster. We found that cluster 3 was characterized by more persistent symptoms in multiple SOCs at 3 months after hospital presentation, while individuals in cluster 1 recovered from their acute symptoms (Supplementary Figure 2 and Supplementary Table 10).

## Discussion

We identified three phenotypic clusters based upon the temporal trajectories of symptoms: remitting, persistent, and incident. Individuals in cluster 1 had a high hospitalization rate, but lower prevalence of PASC in what would be characterized as a “remitting” group (Figure 2C and Table 3). In contrast, in cluster 3, individuals had a lower rate of hospitalization, but incident (new) symptoms and high PASC symptom burden in what would be characterized as an “incident” group. Lastly, individuals in cluster 2 had a high hospitalization rate and a relatively high persistent symptom burden (“persistent” group).

Interestingly, the incident group had a preponderance of symptoms emerging in the SOCs: Nervous system disorders, respiratory, and general disorders (Supplementary Figure 2). In contrast, the remitting group had the least burden of psychiatric conditions when compared to other groups (Supplementary Figure 2). While the finding in the remitting group may indicate greater ability to resolve symptoms associated with infection, the finding of new symptoms in the incident group may suggest an autoimmune phenomenon or viral persistence (Supplementary Figure 2) (11, 12). Additionally, the nature and extent of regenerative or repair mechanisms could conceivably influence symptom evolution and provide an explanation for different temporal patterns in the remitting versus persistent group. Other studies from early in the pandemic have also utilized clustering of symptoms to identify various phenotypes of COVID-19 and PASC (6, 13–15), though the populations analyzed and the timing and types of symptoms collected varied across studies. Although the clusters that we identified in our study may represent only a subset of the total PASC population, and the utility of these clusters for disease management requires further research and validation, it is notable that evidence for heterogeneity in underlying symptoms and mechanisms of PASC continue to emerge (16–19). Taken together, the differences in COVID-19 hospitalization and PASC prevalence in the incident versus remitting groups could point to critical differences in the underlying mechanisms and approaches to preventing and managing PASC. We acknowledge that operational challenges in implementing the study during the pandemic led to the fact that the cluster analysis population did not include all study participants, and was slightly younger, had a greater proportion of women, and a shorter time-period between COVID-19 onset and hospital presentation; therefore, further research is needed to confirm our findings.

The prevalence of PASC in our participants was high (38.7%), but this is comparable to other reports during the pandemic time-period during which the alpha and beta variants were predominant (20–22). Similar to prior reports, the prevalence of PASC in ambulatory care participants was lower than that in hospitalized participants and occurred in older individuals (Table 2) (23), providing external evidence supporting our findings. Most PASC studies from early in the pandemic have often focused on the follow-up of hospitalized patients with COVID-19 (24). The results presented in this report include both hospitalized and non-hospitalized participants and pertain to an early period (May 2020 to June 2021) of the pandemic before proven-effective vaccines, antivirals, and biologics were widely employed. Thus, our study is well-suited to identify clinical insights that merit further investigation about the pathogenesis of PASC resulting from a SARS-CoV-2 infection and the host response early in the pandemic. Our study complements the work underway in the NIH RECOVER Initiative that was launched in 2022 (Trial Registration Number: NCT05172024).

A secondary and intriguing finding was that systemic corticosteroids given during acute COVID-19 infection were strongly associated with PASC at 3 months. While systemic corticosteroids may confer survival advantage during acute illness, there may be an increase in long-term risk for PASC due to immune dysregulation (25), a “*survivorship effect*,” or residual confounding despite efforts to adjust with propensity scores (9). Alternatively, high-dose steroids during acute illness may increase the risk for bacterial superinfection (26), which, in turn, could aggravate organ damage leading to persistence of symptomatology and PASC (3). Similarly, other associations or side effects of systemic corticosteroids (e.g., metabolic alkalosis and GERD) may be associated with the corticosteroid administration or alternatively may merely indicate the presence of multiple comorbidities (2, 27). Considering that high-dose steroids can cause metabolic alkalosis, low lymphocyte count, and GERD, there is biological plausibility that these discoveries are associated through related mechanisms. Importantly, the associations between these clinical factors and PASC should not be interpreted as causal. Rather, they represent areas for further investigation of risk factors and causal mechanisms. Klein et al. (28) have reported that levels of cortisol were uniformly lower among participants with PASC relative to matched control groups. Our finding of association between PASC and systemic corticosteroid administration may indicate the basis for the observed association between PASC and low serum cortisol levels due to suppression of hypothalamic-pituitary-adrenal axis (29).

In summary, our findings from patients with SARS-CoV-2 infection during the early stages of the pandemic emphasize the importance of longitudinal studies aimed at understanding the various PASC trajectories as a key step toward gaining mechanistic insight. Future research is needed to validate our findings in a separate cohort and further characterize individuals with varied disease trajectories by molecular analysis aimed at identifying diagnostic signatures and candidate therapeutic mechanisms for more effective disease management.

## Data availability statement

The datasets presented in this article are not readily available because of patient confidentiality limitations. Requests to access the datasets should be directed to immuneprofiler@verily.com.

## Ethics statement

The studies involving humans were approved by the Study Protocol # 20201016 WCG Institutional Review Board. The studies were conducted in accordance with the local legislation and institutional requirements. The participants provided their written informed consent to participate in this study.

## Author contributions

HZ, WC, and CK conceptualized the study. CC, KD, HZ, WC, VR, MB, and JK were involved in the study design. SP, VT, JM, CRd, IR, MB, MS, and JK recruited patients and collected the samples. CC, SP, JL, MW, and MS analyzed the data. CC, SP, JL, MW, MS, JK, and CK wrote the manuscript. CC, SP, JL, MW, KD, VR, HZ, WC, VT, JM, CRd, IR, BP, MB, MS, JK, and CK reviewed the manuscript. All authors contributed to the article and approved the submitted version.
