# Supplementary material for: Distinct temporal trajectories and risk factors for Post-acute sequelae of SARS-CoV-2 infection
Source: Front Med (Lausanne). 2023 Oct 16;10:1227883. doi: 10.3389/fmed.2023.1227883 (PMC10614284; doi:10.3389/fmed.2023.1227883)
Supplement: Supplementary file 1 [file Data_Sheet_1.docx]

***Supplementary Material***

**Distinct temporal trajectories and risk factors for Post-acute sequelae of SARS-CoV-2 infection**

Chen Chen, Ph.D.,^†^ Sairam Parthasarathy, M.D.,^†^ Jacqueline M. Leung, Ph.D., Michelle J. Wu, Ph.D., Katherine A. Drake, Ph.D., Vanessa K. Ridaura, Ph.D., Howard C. Zisser, M.D., William A. Conrad, M.D., Victor F. Tapson, M.D., James N. Moy, M.D., Christopher R. deFilippi, M.D., Ivan O. Rosas, M.D., Bellur S. Prabhakar, Ph.D., Mujeeb Basit, M.D., Mirella Salvatore, M.D., Jerry A. Krishnan, M.D., Ph.D.,^$^* Charles C. Kim, Ph.D.^$^*

^†^ These authors share first authorship

^$^ These authors share senior authorship

*** Correspondence:**

Corresponding Authors

jakris@uic.edu

charliekim@verily.com

**ACKNOWLEDGEMENTS**

We thank all the participants and study staff that were involved in this study during a challenging time when COVID-19 was first emerging.

| **Acknowledged individual** | **Role** | **Affiliation** |
| --- | --- | --- |
| Benazir Khan, MD | Recruitment and retention of research participants | Baylor College of Medicine |
| Fernando Poli, MD | Recruitment and retention of research participants | Baylor College of Medicine |
| Rafael Cardenas Castillo, MD | Recruitment and retention of research participants | Baylor College of Medicine |
| Antonina Caudill, MPH | Recruitment and retention of research participants, collected data | Cedars-Sinai Medical Center |
| Cristabelle Ojukwu, BS | Sample processing | Cedars-Sinai Medical Center |
| Devin Fine, BS | IRB and regulatory issues | Cedars-Sinai Medical Center |
| Emad Bayoumi, MD, MBA | Recruitment and retention of research participants, collected data | Cedars-Sinai Medical Center |
| Ethan Pascual, MA | Recruitment and retention of research participants, collected data | Cedars-Sinai Medical Center |
| Gregg Clapham, MA | Administrative tasks, Inventory | Cedars-Sinai Medical Center |
| Lisa Herrera | Administrative tasks | Cedars-Sinai Medical Center |
| Millie Gomez, MD | Budget Coordinator | Cedars-Sinai Medical Center |
| Po-En Chen, RN, BSN | Recruitment and retention of research participants, collected data | Cedars-Sinai Medical Center |
| Sara Langley, RN, MSN | Recruitment and retention of research participants, collected data | Cedars-Sinai Medical Center |
| Susan Jackman, RN, MS | Recruitment and retention of research participants, collected data, IRB and regulatory issues, budget and contract coordinator | Cedars-Sinai Medical Center |
| Tabia Richardson, MPH, PhD | Supervised team | Cedars-Sinai Medical Center |
| Treasure Joyce | Logistics organization | Providence Little Company of Mary Medical Center Torrance |
| Amy Gosha, BSN | Recruitment and retention of research participants, collected data | Rush University Medical Center |
| Heidi Erickson, RN | Recruitment and retention of research participants, collected data, IRB and regulatory issues, critically reviewed the study proposal | University of Arizona |
| Anjana Maheswaran | Coordinator | University of Illinois Chicago |
| Dawood Darbar, MBChB, MD | Investigator | University of Illinois Chicago |
| Heather Prendergast, MD | Investigator | University of Illinois Chicago |
| Jan Kitajewski, PhD | Investigator | University of Illinois Chicago |
| Janet Lin, MD | Site PI | University of Illinois Chicago |
| Jeff Jacobson, MD | Investigator | University of Illinois Chicago |
| Jonathan Klein, MD | Investigator | University of Illinois Chicago |
| Julie DeLisa, MS | Director, Regulatory Affairs | University of Illinois Chicago |
| Lauren Castro, RN | Research Nurse and Nurse Manager | University of Illinois Chicago |
| Lourdes Norwick, RN | Research Nurse | University of Illinois Chicago |
| Marina Del Rios, MD | Investigator | University of Illinois Chicago |
| Patricia Finn, MD | Investigator | University of Illinois Chicago |
| Pavitra Kotini-Shah MD | Investigator | University of Illinois Chicago |
| Research staff in the Emergency Department, inpatient units, and laboratories at University of Illinois Chicago | Conducted study | University of Illinois Chicago |
| Richard Novak, MD | Investigator | University of Illinois Chicago |
| Sai Illendula, MS | Data Manager | University of Illinois Chicago |
| Shaveta Khosla | Coordinator | University of Illinois Chicago |
| Terry Vanden Hoek, MD | Investigator | University of Illinois Chicago |
| Wendy Haase, MBA | Project Manager | University of Illinois Chicago |
| Yining Chen | Coordinator | University of Illinois Chicago |
| Richard Medford, MD | Site PI | University of Texas Southwestern Medical Center |
| Samuel McDonald MD, MS | Site PI | University of Texas Southwestern Medical Center |
| Biostatistics Team | Managed and analyzed clinical data | Verily Life Sciences |
| Clinical Data Management team | Co-ordinated sites, managed clinical data | Verily Life Sciences |
| Clinical Operations Team | Co-ordinated sites, managed clinical data | Verily Life Sciences |
| Leera Choi | Co-ordinated sites, contract coordinator, IRB, study initiation | Verily Life Sciences |
| Molecular Production team | Sample processing | Verily Life Sciences |
| Weill Cornell Medicine Clinical & Translational Science Center | Conducted study | Weill Cornell Medicine Clinical & Translational Science Center |
| Anna Gwak, BA | IRB and regulatory issues | Weill Cornell Medicine Transplantation-Oncology Infectious Diseases Clinical Research Unit |
| Elizabeth Salsgiver, BS, MPH | Budget and contract coordinator, logistics organization | Weill Cornell Medicine Transplantation-Oncology Infectious Diseases Clinical Research Unit |
| Jack Spagnoletti, BA | Recruitment and retention of research participants, collected data | Weill Cornell Medicine Transplantation-Oncology Infectious Diseases Clinical Research Unit |

**SUPPLEMENTARY METHODS**

**Study Oversight**

The Predictors of Severe COVID-19 Outcomes (PRESCO) study was conducted at eight institutions across the United States and was approved by a central WCG Institutional Review Board (WCG IRB Protocol # 20201016) and each academic institution (Trial Registration Number: NCT04388813). The PRESCO study was designed as a longitudinal, multi-center, observational study collecting diverse biological measurements and clinical and epidemiological data for the purpose of enabling a greater understanding of the onset of severe outcomes, primarily acute respiratory distress syndrome (ARDS) and/or mortality, in patients presenting to the hospital with suspicion or diagnosis of COVID-19. We sought to understand whether there were early signatures that predict progression to ARDS, mortality, and/or other comorbid conditions. The duration of the study participation was approximately 3 months. When the existence of long-term consequences of acute SARS-CoV-2 infection became apparent, the 3-month outcomes collection was amended to look at post-acute sequelae of SARS-CoV-2 infection (PASC). Due to the delayed implementation of the amendment, PASC information and 3-month follow-up symptoms were not collected from participants who exited the study before May 2021. Resource constrained data retention from exited participants and missing data were not imputed given the observational nature of the study. The WCG IRB Protocol number was utilized by all sites unless stated otherwise (The University of Arizona, Cedars-Sinai Medical Center, University of Illinois at Chicago, Rush University Medical Center (IRB #20050404-IRB02), Weill Cornell Medical College (IRB #20-08022553), University of Texas Southwestern Medical Center, Baylor College of Medicine, and Inova Health Care Services). All participants or their legally authorized representatives provided written informed consent.

**Participant Recruitment**

Participants were recruited into the PRESCO study from hospitalized and non-hospitalized (ambulatory) settings. For hospitalized patients, a partial HIPAA waiver from the Institutional Review Board was obtained at all of the sites. Clinical research coordinators in the PRESCO study assessed new admission patient lists and from these patients, selected those that were COVID-19 positive cases. After receiving permission from the attending physician, patients, and/or their surrogates (in the event that the potential participant was unable to give informed consent), participants were approached by the clinical research coordinators, who explained the purpose and risks for participation in the PRESCO study as part of the informed consenting process. For ambulatory patients, flyers regarding the PRESCO study were posted in urgent care facilities, COVID-19 testing centers, and ambulatory clinics. These flyers indicated the nature and purpose of the PRESCO study. Potential patients who tested positive for SARS-CoV-2 infection called the clinical research coordinators’ phone numbers that were listed on the flyers. Additionally, providers who were caring for patients in clinics and other facilities could also refer potential participants to the study if their patients exhibited symptoms of COVID-19 and tested positive.

**Measures**

At enrollment (Visit 1), demographic data such as participants’ age, sex, race, ethnicity, and state of residence were collected, in addition to clinical data regarding the participants’ height, weight, vital signs (blood pressure, temperature, pulse oximetry, and capnography), medical history and concurrent medical events (comorbidities; alcohol, tobacco, and caffeine use; COVID-19 diagnosis’ relevant symptoms, and risk factors; onset, severity, and resolution of ARDS; and mortality), prior and concomitant medications, hospitalization details (inpatient surgeries or procedures, including details of mechanical ventilation, and duration of hospitalization), standard clinical laboratory tests, and research biospecimens were also collected. A COVID-19 epidemiological and clinical questionnaire was administered at Visit 1. During Visit 1, participants also selected symptoms that were present from a list of 22 symptoms. Symptoms were grouped and analyzed by System Organ Class (SOC) according to the Medical Dictionary for Regulatory Activities (MedDRA) (**Supplementary Table 1**), which groups symptoms by etiology, manifestation site, and/or purpose.

If one was admitted to the hospital and/or the intensive care unit (ICU), at the associated study visits (hospital admission - Visit 2, ICU admission - Visit 3, and hospital discharge - Visit 4), clinical laboratory tests, research biospecimens, vital signs, concomitant medications, and hospitalization details were collected.

At the 3-month follow up visit (Visit 5), besides clinical laboratory tests, research biospecimens, vital signs, and concomitant medications, an additional follow-up survey was given to each participant to collect health-related information after discharge. WHO scale was used to measure COVID-19 severity. At 3 months, participants were asked to select from a list of 30 symptoms if they experienced any persistent symptoms since their last study visit (**Supplementary Table 2**). These symptoms were also grouped and analyzed by SOC according to MedDRA.

**Statistical Analysis**

**Analysis Objectives**

The primary objective of the manuscript is to search for clinical markers that are associated with PASC. The exploratory objective is to identify clinical phenotypes of SARS-CoV-2.

**Sample Size Consideration**

Sample size calculation was not performed given the observational and exploratory nature of the analysis. All available data were used in the analysis. Missing data were not imputed and analyses were conducted on complete cases.

**Analysis Population**

Three analysis populations are defined in the manuscript, enrolled population, PASC analysis population, and cluster analysis population.

- The enrolled population included patients who signed the informed consent and were enrolled in the study.
- The PASC analysis population included patients who had sufficient data for determination of whether they were in the PASC or non-PASC group. Patients with sufficient data were those who responded to the outcome survey on the question, “After your last study visit, how many weeks passed until you were feeling well/normal?”.
- The cluster analysis population included patients who reported symptoms in the outcome survey at the 3-month follow-up visit.

The enrolled population was used in summary of demographic and clinical characteristics of the entire PRESCO cohort. The PASC analysis population was used in PASC association analysis and the cluster analysis population was used in the clustering analysis.

**Analysis Method**

General

Continuous data were summarized by n (count of patients with data available), mean or median, and standard deviation (SD) or minimum and maximum, as appropriate. Categorical data were summarized by frequency and percentage. Pairwise univariate testing of continuous variables (e.g. age, hospital duration) was conducted using the Wilcoxon rank sum test. Pairwise univariate testing of categorical variables with two levels (e.g. sex and concomitant medications) were conducted using Fisher’s exact test and Chi-square test was used in tests with more than two levels (e.g. race and site).

Analysis for the Primary Objective: PASC association analysis

Univariate analysis:

- Unadjusted univariate tests were conducted for all demographic information, clinical characteristics, and clinical labs to search for statistically significant differences between PASC and non-PASC groups. Standardized effect size and p-values were reported.
- Comorbidities that were found significant in univariate testing were reevaluated adjusting for age, sex, whether one has BMI ≥30 kg/m^2^, race-ethnic group, and WHO scale. Concomitant medications that were found to be significantly associated with PASC were reevaluated after adjusting for propensity scores and highest COVID severity (to account for indication bias). Propensity scores were computed from variables derived from the literature to increase the probability of a participant to receive the medication for COVID. All clinical labs at Visit 1 were reevaluated after adjusting for participants’ age and COVID severity evaluated at Visit 1, and those at Visit 4 and Visit 5 were reevaluated adjusting for participants’ age. Logistic regressions were used to conduct the association analysis after adjusting for confounders.
- Standardized effect size of continuous measures was estimated by Cohen's d and standardized effect size of proportions was taken by taking angular transformation of each proportion following Cohen (1988) (1), and effect size of odds ratio from logistic regression was taken by following Sanchez-Meca et. al. (2003) equation 15 (2).

Multivariable analysis: Multivariable analysis was conducted for clinical markers that were identified significant in the above univariate analysis. Forward stepwise logistic regressions were run with age and WHO scale forced into the model selection process. Akaike Information Criterion (AIC) was used as the selection criteria.

Analysis for the Exploratory Objective: clustering analysis

*Identification of clusters*: Participants that provided information regarding the specific long-term symptoms of PASC at the follow-up visit approximately 3 months after hospital presentation were clustered based on a binary (yes/no) report of their persisting symptoms. Hamming distance was used as the distance metric, and hierarchical clustering of participants was performed with Ward's method. The dendrogram was then cut at an appropriate height to generate three resulting clusters.

*Evaluation of clusters:* Pairwise tests were conducted to test statistical differences in demographics information, clinical characteristics, and clinical laboratory results. The pairwise tests were done for each pair of clusters identified in the above step. Symptoms collected at enrollment and 3-month follow up and their mapped SOC were summarized descriptively for each cluster. Symptoms were mapped to a SOC according to MedDRA. Symptom evolutions for each cluster were summarized by SOC. Specifically, based on a patient’s experience of a given SOC at enrollment and 3-month follow up, their symptom evolution can be categorized as never experienced, new onset, recovered, persistent.

**Multiplicity consideration**

Multiplicity was corrected for analyses of the primary objective, i.e. PASC association analysis. Specifically, univariate tests of association with PASC in comorbidities, concomitant medications, and clinical labs were corrected. The Bejamini-Hochberg method was used for multiplicity control at FDR=0.05 for each set of analysis.

**Missing data consideration**

Due to the delayed implementation of the amendment, PASC information and 3-month follow-up symptoms were not collected from participants who exited the study before May 2021. Resource constrained data retention from exited participants and missing data were not imputed given the observational nature of the study. All analyses were based on complete cases.

**Analysis Software**

The hierarchical clustering analysis was conducted using the *pheatmap* package and the multivariable analysis was conducted using the *step()* function in the BASE package of the R language. The remaining analyses were conducted in Python with modules including *pandas, numpy, statsmodels, scipy,* and *sklearn*.

**Definition of Key Variables**

| **Key variables** | **Definition** |
| --- | --- |
| COVID start date | COVID start date is defined as the minimum of a patient’s self reported first positive test date, enrollment date, hospital presentation date, and hospitalization date. |
| COVID severity | COVID severity is evaluated by WHO scale (3) |
| PASC | A patient is defined as PASC if they are reported to have taken 4 or more weeks after their COVID start date to recover to their usual state of health. |

**References for Statistical Analysis**

1. [Cohen J. Statistical Power Analysis for the Behavioral Sciences. 2nd Edition. Routledge; 1988.](http://paperpile.com/b/Nxx3Us/EPSE)
2. [Sánchez-Meca J, Marín-Martínez F, Chacón-Moscoso S. Effect-size indices for dichotomized outcomes in meta-analysis. Psychol Methods 2003;8:448–67.](http://paperpile.com/b/Nxx3Us/7n3p)
3. World Health Organization. WHO R&D Blueprint novel Coronavirus COVID-19 Therapeutic Trial Synopsis. 2022; Available from: https://www.who.int/docs/default-source/blue-print/covid-19-therapeutic-trial-synopsis.pdf

**SUPPLEMENTARY FIGURES**

**Supplementary Figure 1:** PRESCO enrollment ran from May 2020 until June 2021, with peak enrollment during the end of the 2020 through the beginning of 2021.


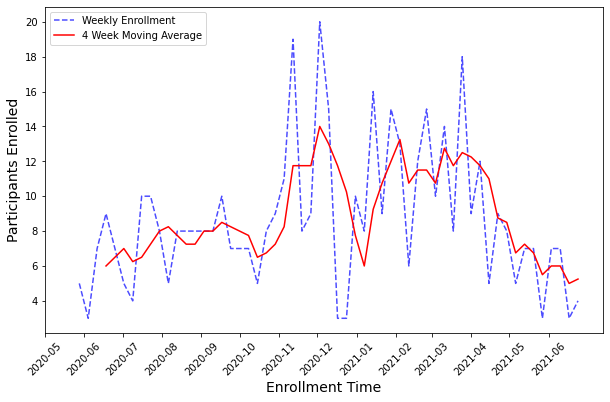


**Supplementary Figure 2:** This figure shows the evolution of symptomatology from enrollment to the 3-month follow-up visit (Visit 5), categorized by system organ class (SOC) and cluster number. For each SOC, participants are categorized into four groups based on whether or not they had any symptoms in that class at enrollment and at Visit 5.


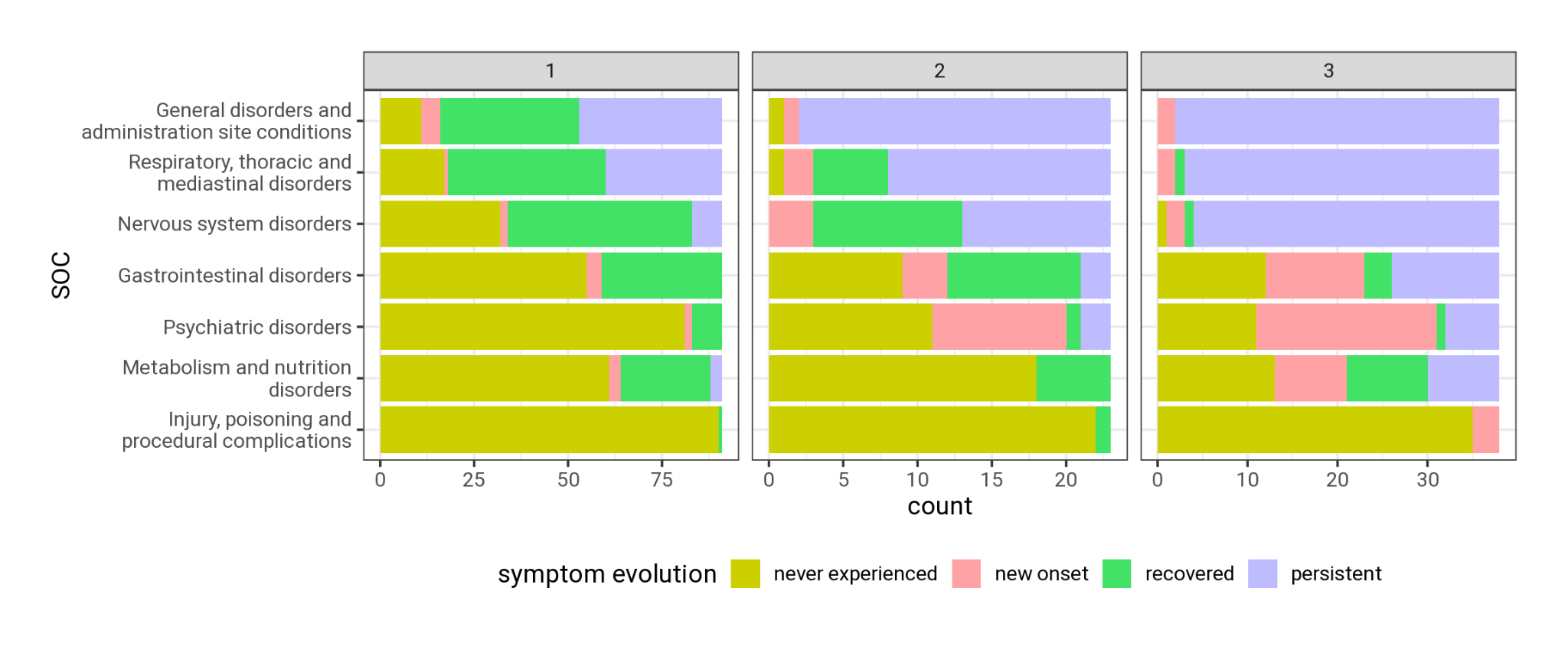


**SUPPLEMENTARY TABLES**

**Supplementary Table 1:** At enrollment, each participant was asked "In the last 14 days before receiving care at the participating hospital, have you had any of the following symptoms? Select all that apply." This table shows the symptoms listed, the System Organ Class (SOC) classification based on MedDRA for each, and the counts and percentages for each analysis population.

| **Symptoms** | **System Organ Class** | **Enrolled population**  **(N=494)** | **Patients with PASC information collected**  **(PASC analysis population)**  **(N=354)** | **Patients with symptoms collected at the 3-month follow-up visit (Visit 5)**  **(Cluster analysis population)**  **(N=152)** |
| --- | --- | --- | --- | --- |
| Abdominal pain | Gastrointestinal disorders | 112 (22.8) | 71 (20.1) | 31 (20.4) |
| Gastrointestinal symptoms (e.g., upset stomach, nausea) | Gastrointestinal disorders | 193 (39.2) | 130 (36.7) | 43 (28.3) |
| Vomiting | Gastrointestinal disorders | 102 (20.7) | 75 (21.2) | 26 (17.1) |
| Body aches | General disorders and administration site conditions | 260 (52.8) | 196 (55.4) | 85 (55.9) |
| Chest pain / tightness | General disorders and administration site conditions | 178 (36.2) | 127 (35.9) | 60 (39.5) |
| Fatigue | General disorders and administration site conditions | 324 (65.9) | 240 (67.8) | 104 (68.4) |
| Feeling feverish | General disorders and administration site conditions | 237 (48.2) | 168 (47.5) | 69 (45.4) |
| Measured fever (above 100F) | General disorders and administration site conditions | 223 (45.3) | 156 (44.1) | 64 (42.1) |
| Painful red toes (chilblains) | Injury, poisoning and procedural complications | 7 (1.4) | 6 (1.7) | 2 (1.3) |
| Skipping meals / loss of appetite | Metabolism and nutrition disorders | 203 (41.3) | 148 (41.8) | 49 (32.2) |
| A headache of unknown cause | Nervous system disorders | 263 (53.5) | 198 (55.9) | 92 (60.5) |
| Dizziness | Nervous system disorders | 169 (34.3) | 132 (37.3) | 51 (33.6) |
| Loss of smell | Nervous system disorders | 150 (30.5) | 116 (32.8) | 49 (32.2) |
| Loss of taste | Nervous system disorders | 180 (36.6) | 136 (38.4) | 52 (34.2) |
| Confusion / disorientation | Psychiatric disorders | 65 (13.2) | 53 (15.0) | 18 (11.8) |
| Bloody cough or mucus | Respiratory, thoracic and mediastinal disorders | 29 (5.9) | 24 (6.8) | 8 (5.3) |
| Congestion of unknown cause | Respiratory, thoracic and mediastinal disorders | 106 (21.5) | 81 (22.9) | 43 (28.3) |
| Dry cough | Respiratory, thoracic and mediastinal disorders | 284 (57.7) | 205 (57.9) | 89 (58.6) |
| Hoarse voice | Respiratory, thoracic and mediastinal disorders | 105 (21.3) | 79 (22.3) | 39 (25.7) |
| Increased respiratory rate (hyperventilation) | Respiratory, thoracic and mediastinal disorders | 86 (17.5) | 66 (18.6) | 21 (13.8) |
| Shortness of breath | Respiratory, thoracic and mediastinal disorders | 322 (65.4) | 224 (63.3) | 91 (59.9) |
| Wet cough | Respiratory, thoracic and mediastinal disorders | 134 (27.2) | 99 (28.0) | 44 (28.9) |
| None of the above | N/A | 21 (4.3) | 20 (5.6) | 9 (5.9) |

**Supplementary Table 2:** At Visit 5, each participant was asked "After your last study visit, did you experience any of the following symptoms? Select any that apply." This table shows the symptoms listed, the SOC classification for each, and the counts and percentages reported.

| **Symptom** | **System Organ Class** | **Patients with symptoms collected at the 3-month follow-up visit (Visit 5)**  **(Cluster analysis population)**  **(N=152)**  **n (%)** |
| --- | --- | --- |
| Fatigue (tiredness) | General disorders and administration site conditions | 94 (61.8) |
| Shortness of breath (difficulty breathing) | Respiratory, thoracic and mediastinal disorders | 56 (36.8) |
| Physical weakness | General disorders and administration site conditions | 47 (30.9) |
| Cough | Respiratory, thoracic and mediastinal disorders | 46 (30.3) |
| Muscle aches/pains | Musculoskeletal and connective tissue disorders | 45 (29.6) |
| Body aches/pains | General disorders and administration site conditions | 44 (28.9) |
| Headache | Nervous system disorders | 42 (27.6) |
| Problems thinking clearly/brain fog | Psychiatric disorders | 39 (25.7) |
| Feeling generally unwell | General disorders and administration site conditions | 36 (23.7) |
| Decreased sense of smell | Nervous system disorders | 35 (23) |
| Nasal Congestion (stuffy nose) | Respiratory, thoracic and mediastinal disorders | 33 (21.7) |
| Decreased sense of taste | Nervous system disorders | 30 (19.7) |
| Runny nose | Respiratory, thoracic and mediastinal disorders | 27 (17.8) |
| Chest pain/pressure/tightness | General disorders and administration site conditions | 24 (15.8) |
| Chest congestion (mucus in chest) | Respiratory, thoracic and mediastinal disorders | 23 (15.1) |
| Loss of appetite | Metabolism and nutrition disorders | 22 (14.5) |
| Wheezing (whistling sound while breathing) | Respiratory, thoracic and mediastinal disorders | 21 (13.8) |
| Chills | General disorders and administration site conditions | 20 (13.2) |
| Fever (≥ 38.0 ºC or 100.4 ºF) | General disorders and administration site conditions | 20 (13.2) |
| Nausea | Gastrointestinal disorders | 16 (10.5) |
| Diarrhea | Gastrointestinal disorders | 16 (10.5) |
| Sneezing | Respiratory, thoracic and mediastinal disorders | 16 (10.5) |
| Feeling faint | Nervous system disorders | 15 (9.9) |
| Sore throat | Respiratory, thoracic and mediastinal disorders | 15 (9.9) |
| Eyes irritation/discharge | Eye disorders | 13 (8.6) |
| Abdominal/stomach pain | Gastrointestinal disorders | 13 (8.6) |
| Skin rash | Skin and subcutaneous tissue disorders | 9 (5.9) |
| Uncontrollable body shaking/shivering | General disorders and administration site conditions | 6 (3.9) |
| Vomiting | Gastrointestinal disorders | 4 (2.6) |
| Red or bruised looking feet or toes | Injury, poisoning and procedural complications | 3 (2) |
| Other | N/A | 11 (7.2) |
| None of the above - I feel back to my pre-COVID self | N/A | 30 (19.7) |

**Supplementary Table 3:** Comorbidities and symptoms reported in participants with GERD are shown, stratified by PASC.

|  | **PASC**  **(N=15)** | **non-PASC**  **(N=6)** |
| --- | --- | --- |
| Other comorbidities |  |  |
| Hypertension | 9 (60.0) | 6 (100.0) |
| Hyperlipidemia | 5 (33.3) | 3 (50.0) |
| Cesarean section | 4 (26.7) | 2 (33.3) |
| Asthma | 6 (40.0) | 0 |
| Diabetes mellitus | 3 (20.0) | 3 (50.0) |
| Depression | 5 (33.3) | 1 (16.7) |
| Anxiety | 4 (26.7) | 1 (16.7) |
| Sleep apnoea syndrome | 4 (26.7) | 0 |
| Hypothyroidism | 3 (20.0) | 1 (16.7) |
| Anemia | 1 (6.7) | 3 (50.0) |
| Symptoms at Visit 5 |  |  |
| Number of participants who reported symptoms | 10 | 3 |
| Fatigue (tiredness) | 9 (90.0) | 0 |
| Shortness of breath (difficulty breathing) | 7 (70.0) | 0 |
| Problems thinking clearly/brain fog | 6 (60.0) | 0 |
| Abdominal/stomach pain | 4 (40.0) | 0 |
| Muscle aches/pains | 4 (40.0) | 0 |
| Feeling generally unwell | 4 (40.0) | 0 |
| Headache | 4 (40.0) | 0 |
| Decreased sense of taste | 4 (40.0) | 0 |
| Decreased sense of smell | 4 (40.0) | 0 |
| Diarrhea | 3 (30.0) | 0 |

**Supplementary Table 4:** Clinical labs values for PASC and non-PASC groups are shown, along with effect sizes and p-values associated with testing for differences between the two groups (see Methods for details).

| **Visit** | **Clinical lab** | **PASC**  **(N=137)** | **Non-PASC**  **(N=217)** | **Effect Size** | **P-**  **value** | **FDR corrected p-value** | **Covariates adjusted effect size** | **Covariates adjusted p-value** | **Covariates adjusted FDR corrected p-value** |
| --- | --- | --- | --- | --- | --- | --- | --- | --- | --- |
| Visit 1 | Albumin -- g/dL | 124, 3.6 (1.9, 5.0) | 163, 3.6 (1.6, 5.1) | -0.131 | 0.1943 | 0.3756 | 0.0562 | 0.4724 | 0.7515 |
| Visit 1 | C-reactive protein (CRP) -- mg/L | 107, 52.6 (1.6, 370.6) | 143, 36.0 (1.3, 800.0) | 0.019 | 0.0549 | 0.1921 | -0.0854 | 0.3010 | 0.7024 |
| Visit 1 | CBC - Absolute Lymphocyte Count -- 10^3/uL | 118, 0.895 (0.15, 4.51) | 167, 1.29 (0.18, 89.0) | -0.153 | 0.0001 | 0.0047 | -0.9321 | 0.0885 | 0.5165 |
| Visit 1 | CBC - Absolute Neutrophil Count -- 10^3/uL | 113, 4.02 (0.99, 64.4) | 153, 3.9 (0.8, 91.5) | -0.204 | 0.8694 | 0.9221 | -0.1913 | 0.0678 | 0.4744 |
| Visit 1 | CBC - Hematocrit (HCT) -- % | 131, 39.5 (24.2, 58.6) | 195, 39.4 (19.7, 50.8) | 0.153 | 0.2815 | 0.4373 | 0.0918 | 0.1644 | 0.5444 |
| Visit 1 | CBC - Hemoglobin (HGB) -- g/dL | 131, 13.1 (7.9, 18.7) | 195, 13.0 (6.3, 17.7) | 0.154 | 0.2893 | 0.4373 | 0.0888 | 0.1749 | 0.5444 |
| Visit 1 | CBC - Platelet Count (PLT) -- 10^3/uL | 131, 228.0 (45.0, 507.0) | 194, 235.0 (78.0, 667.0) | -0.097 | 0.7104 | 0.8288 | -0.0289 | 0.6544 | 0.8233 |
| Visit 1 | CBC - Red Blood Cell Count (RBC) -- 10^6/uL | 129, 4.5 (2.36, 6.58) | 195, 4.61 (2.57, 10.71) | -0.062 | 0.6610 | 0.7977 | -0.0257 | 0.6935 | 0.8233 |
| Visit 1 | CBC - White Blood Cell Count (WBC) -- 10^3/uL | 131, 6.1 (1.7, 16.09) | 195, 6.05 (1.9, 21.44) | -0.152 | 0.2707 | 0.4373 | -0.1096 | 0.1079 | 0.5394 |
| Visit 1 | Creatinine Kinase -- U/L | 85, 85.0 (13.0, 1279.0) | 109, 72.0 (0.7, 1465.0) | 0.202 | 0.2146 | 0.3756 | 0.0802 | 0.3491 | 0.7090 |
| Visit 1 | D-Dimer -- ug/mL | 98, 0.63 (0.185, 20.0) | 145, 0.55 (0.1, 20.72) | 0.169 | 0.0832 | 0.2149 | 0.0812 | 0.2957 | 0.7024 |
| Visit 1 | Ferritin -- ng/mL | 103, 437.74 (36.0, 7952.0) | 145, 299.0 (7.0, 13152.0) | 0.13 | 0.0299 | 0.1455 | 0.0379 | 0.6027 | 0.8233 |
| Visit 1 | Lactate dehydrogenase -- U/L | 91, 304.0 (108.0, 910.0) | 131, 273.0 (99.0, 1908.0) | 0.157 | 0.0333 | 0.1455 | 0.0000 | 0.9999 | 0.9999 |
| Visit 1 | Liver Function Test - ALT (SGPT) -- U/L | 112, 30.0 (4.0, 330.0) | 160, 25.0 (6.0, 434.0) | 0.076 | 0.0196 | 0.1371 | 0.0271 | 0.6991 | 0.8233 |
| Visit 1 | Liver Function Test - AST (SGOT) -- U/L | 114, 33.0 (8.0, 200.0) | 160, 27.0 (10.0, 572.0) | 0.052 | 0.0168 | 0.1371 | -0.0077 | 0.9108 | 0.9541 |
| Visit 1 | Liver Function Test - Alkaline Phosphatase -- U/L | 123, 70.0 (28.0, 243.0) | 169, 74.0 (25.0, 370.0) | -0.085 | 0.5475 | 0.7097 | -0.0603 | 0.3849 | 0.7090 |
| Visit 1 | Liver Function Test - Bilirubin, Direct -- mg/dL | 83, 0.2 (0.0, 1.0) | 124, 0.2 (0.0, 1.0) | -0.275 | 0.1729 | 0.3560 | -0.1936 | 0.0440 | 0.3849 |
| Visit 1 | Liver Function Test - Bilirubin, Total -- mg/dL | 125, 0.4 (0.1, 5.2) | 167, 0.5 (0.2, 8.1) | -0.172 | 0.0817 | 0.2149 | -0.1317 | 0.1951 | 0.5444 |
| Visit 1 | Liver Function Test - Protein, Total -- g/dL | 125, 6.9 (3.7, 8.3) | 166, 7.0 (4.6, 51.0) | -0.149 | 0.1349 | 0.3147 | -0.1215 | 0.4679 | 0.7515 |
| Visit 1 | Partial Thromboplastin Time (PTT) -- seconds | 87, 29.9 (1.1, 47.0) | 123, 31.0 (1.2, 168.0) | -0.231 | 0.0293 | 0.1455 | -0.4234 | 0.0261 | 0.3849 |
| Visit 1 | Procalcitonin -- ng/mL | 87, 0.07 (0.01, 8.26) | 118, 0.05 (0.0, 10.32) | 0.106 | 0.0042 | 0.0737 | 0.0432 | 0.5859 | 0.8233 |
| Visit 1 | Prothrombin Time (PT) -- seconds | 97, 12.9 (10.0, 23.0) | 133, 13.1 (9.8, 26.4) | -0.165 | 0.2999 | 0.4373 | -0.2133 | 0.0199 | 0.3849 |
| Visit 1 | Renal Function Test - BUN -- mg/dL | 131, 14.0 (5.0, 85.0) | 194, 14.0 (4.0, 98.0) | 0.116 | 0.3921 | 0.5490 | -0.0060 | 0.9269 | 0.9541 |
| Visit 1 | Renal Function Test - BUN/Creatinine Ratio | 86, 16.15 (6.3, 42.0) | 103, 14.7 (1.9, 43.8) | 0.235 | 0.0860 | 0.2149 | 0.0258 | 0.7710 | 0.8705 |
| Visit 1 | Renal Function Test - Calcium -- mg/dL | 129, 8.6 (5.9, 98.0) | 191, 8.6 (7.2, 10.8) | 0.135 | 0.8233 | 0.9221 | 0.5428 | 0.3610 | 0.7090 |
| Visit 1 | Renal Function Test - Carbon Dioxide, Total -- mmol/L | 128, 24.0 (15.0, 35.0) | 184, 23.0 (17.0, 32.0) | 0.102 | 0.4117 | 0.5542 | 0.0836 | 0.2022 | 0.5444 |
| Visit 1 | Renal Function Test - Chloride -- mmol/L | 130, 103.0 (94.0, 113.0) | 193, 104.0 (91.0, 114.0) | -0.216 | 0.0150 | 0.1371 | -0.0943 | 0.1447 | 0.5444 |
| Visit 1 | Renal Function Test - Creatinine -- mg/dL | 131, 0.81 (0.36, 7.03) | 193, 0.8 (0.08, 10.44) | -0.11 | 0.9889 | 0.9889 | -0.0938 | 0.2001 | 0.5444 |
| Visit 1 | Renal Function Test - Glucose -- mg/dL | 131, 118.0 (50.0, 479.0) | 194, 109.5 (0.6, 419.0) | 0.068 | 0.0692 | 0.2149 | -0.0250 | 0.7057 | 0.8233 |
| Visit 1 | Renal Function Test - Phosphorus -- mg/dL | 82, 3.1500000000000004 (1.5, 4.8) | 95, 3.2 (0.9, 6.2) | -0.113 | 0.6418 | 0.7977 | -0.0764 | 0.3778 | 0.7090 |
| Visit 1 | Renal Function Test - Potassium -- mmol/L | 130, 4.05 (3.0, 5.5) | 193, 4.1 (3.2, 6.6) | -0.161 | 0.2099 | 0.3756 | -0.1409 | 0.0378 | 0.3849 |
| Visit 1 | Renal Function Test - Sodium -- mmol/L | 130, 138.0 (128.0, 146.0) | 194, 138.0 (127.0, 146.0) | -0.13 | 0.1545 | 0.3379 | -0.0276 | 0.6700 | 0.8233 |
| Visit 1 | Renal Function Test - eGFR If African American -- mL/min/1.73 | 34, 87.0 (7.2, 176.0) | 63, 87.0 (7.0, 194.9) | -0.022 | 0.9307 | 0.9581 | 0.0894 | 0.5180 | 0.7882 |
| Visit 1 | Renal Function Test - eGFR If NonAfrican American -- mL/min/1.73 | 51, 88.9 (14.0, 148.0) | 68, 89.0 (4.9, 145.9) | -0.002 | 0.8549 | 0.9221 | 0.0290 | 0.8043 | 0.8798 |
| Visit 1 | Troponin -- ng/mL | 59, 0.01 (0.0, 0.4) | 99, 0.01 (0.0, 1.73) | -0.147 | 0.0374 | 0.1455 | -0.1739 | 0.4347 | 0.7515 |
| Visit 4 | Albumin -- g/dL | 53, 3.2 (1.3, 4.1) | 46, 3.1 (1.8, 4.4) | 0.025 | 0.7146 | 0.9595 | 0.0279 | 0.8120 | 0.9235 |
| Visit 4 | C-reactive protein (CRP) -- mg/L | 38, 17.700000000000003 (2.0, 114.0) | 34, 26.6 (2.0, 161.2) | -0.225 | 0.4703 | 0.9595 | -0.1229 | 0.3717 | 0.9101 |
| Visit 4 | CBC - Absolute Lymphocyte Count -- 10^3/uL | 55, 1.5 (0.4, 4.2) | 57, 1.59 (0.3, 4.9) | -0.053 | 0.6582 | 0.9595 | 0.0334 | 0.7710 | 0.9235 |
| Visit 4 | CBC - Absolute Neutrophil Count -- 10^3/uL | 52, 5.715 (2.26, 58.9) | 53, 5.56 (1.86, 68.3) | -0.195 | 0.8400 | 0.9595 | -0.1174 | 0.3481 | 0.9101 |
| Visit 4 | CBC - Hematocrit (HCT) -- % | 68, 39.8 (22.5, 58.6) | 77, 39.1 (19.8, 47.5) | 0.196 | 0.3769 | 0.9248 | 0.0924 | 0.3358 | 0.9101 |
| Visit 4 | CBC - Hemoglobin (HGB) -- g/dL | 68, 12.9 (7.9, 19.1) | 77, 12.9 (7.1, 15.6) | 0.184 | 0.5132 | 0.9595 | 0.0842 | 0.3776 | 0.9101 |
| Visit 4 | CBC - Platelet Count (PLT) -- 10^3/uL | 68, 290.5 (28.0, 806.0) | 77, 280.0 (5.9, 695.0) | 0.017 | 0.7214 | 0.9595 | 0.0299 | 0.7503 | 0.9235 |
| Visit 4 | CBC - Red Blood Cell Count (RBC) -- 10^6/uL | 68, 4.505 (2.28, 6.7) | 77, 4.56 (2.16, 5.71) | 0.104 | 0.8710 | 0.9595 | 0.0404 | 0.6692 | 0.9101 |
| Visit 4 | CBC - White Blood Cell Count (WBC) -- 10^3/uL | 68, 8.405000000000001 (4.2, 16.29) | 77, 8.4 (3.44, 19.04) | -0.133 | 0.7288 | 0.9595 | -0.0672 | 0.4728 | 0.9101 |
| Visit 4 | Creatinine Kinase -- U/L | 33, 40.0 (12.0, 775.0) | 25, 71.0 (11.0, 891.0) | -0.153 | 0.1575 | 0.6181 | -0.0884 | 0.5510 | 0.9101 |
| Visit 4 | D-Dimer -- ug/mL | 44, 0.6 (0.15, 13.34) | 35, 0.7 (0.00048, 15.82) | -0.111 | 0.4958 | 0.9595 | -0.0679 | 0.5971 | 0.9101 |
| Visit 4 | Ferritin -- ng/mL | 45, 403.0 (50.0, 2745.1) | 31, 490.4 (11.0, 5094.0) | -0.196 | 0.8533 | 0.9595 | -0.1000 | 0.4465 | 0.9101 |
| Visit 4 | Lactate dehydrogenase -- U/L | 42, 286.5 (144.0, 651.0) | 24, 320.0 (158.0, 644.0) | -0.132 | 0.7139 | 0.9595 | -0.0648 | 0.6499 | 0.9101 |
| Visit 4 | Liver Function Test - ALT (SGPT) -- U/L | 47, 45.0 (6.0, 257.0) | 50, 37.0 (8.0, 329.0) | 0.013 | 0.8995 | 0.9595 | 0.0144 | 0.9004 | 0.9383 |
| Visit 4 | Liver Function Test - AST (SGOT) -- U/L | 49, 28.0 (7.0, 265.0) | 50, 29.0 (9.0, 101.0) | -0.02 | 0.6044 | 0.9595 | -0.0064 | 0.9547 | 0.9547 |
| Visit 4 | Liver Function Test - Alkaline Phosphatase -- U/L | 56, 73.5 (26.0, 255.0) | 52, 71.0 (40.0, 212.0) | 0.035 | 0.9681 | 0.9681 | 0.0213 | 0.8420 | 0.9235 |
| Visit 4 | Liver Function Test - Bilirubin, Direct -- mg/dL | 37, 0.21 (0.0, 0.6) | 38, 0.2 (0.0, 2.5) | -0.249 | 0.8635 | 0.9595 | -0.1675 | 0.3984 | 0.9101 |
| Visit 4 | Liver Function Test - Bilirubin, Total -- mg/dL | 56, 0.5 (0.2, 3.1) | 53, 0.5 (0.2, 5.5) | -0.272 | 0.5512 | 0.9595 | -0.1647 | 0.2162 | 0.9101 |
| Visit 4 | Liver Function Test - Protein, Total -- g/dL | 56, 6.35 (5.0, 8.1) | 53, 6.4 (4.3, 7.7) | 0.025 | 0.9298 | 0.9595 | 0.0255 | 0.8128 | 0.9235 |
| Visit 4 | Partial Thromboplastin Time (PTT) -- seconds | 31, 27.4 (22.3, 52.3) | 18, 30.3 (26.3, 40.6) | -0.291 | 0.0390 | 0.6181 | -0.1870 | 0.2753 | 0.9101 |
| Visit 4 | Procalcitonin -- ng/mL | 25, 0.07 (0.01, 0.64) | 15, 0.19 (0.01, 1.11) | -0.743 | 0.1613 | 0.6181 | -0.4666 | 0.0586 | 0.9101 |
| Visit 4 | Prothrombin Time (PT) -- seconds | 38, 11.6 (10.0, 26.8) | 27, 13.5 (10.5, 24.6) | -0.296 | 0.1100 | 0.6181 | -0.1870 | 0.2032 | 0.9101 |
| Visit 4 | Renal Function Test - BUN -- mg/dL | 70, 19.0 (6.0, 98.0) | 76, 17.0 (3.0, 69.0) | 0.175 | 0.1719 | 0.6181 | 0.0479 | 0.6307 | 0.9101 |
| Visit 4 | Renal Function Test - BUN/Creatinine Ratio | 52, 22.25 (11.3, 40.9) | 34, 17.85 (2.9, 58.0) | 0.241 | 0.0842 | 0.6181 | 0.1403 | 0.3097 | 0.9101 |
| Visit 4 | Renal Function Test - Calcium -- mg/dL | 69, 8.6 (7.0, 11.0) | 75, 8.5 (7.0, 90.0) | -0.135 | 0.1210 | 0.6181 | -0.0978 | 0.5719 | 0.9101 |
| Visit 4 | Renal Function Test - Carbon Dioxide, Total -- mmol/L | 69, 25.0 (8.7, 35.0) | 71, 23.0 (17.0, 33.0) | 0.476 | 0.0013 | 0.0458 | 0.2713 | 0.0099 | 0.3351 |
| Visit 4 | Renal Function Test - Chloride -- mmol/L | 69, 104.0 (93.0, 114.0) | 76, 104.0 (88.0, 112.0) | -0.095 | 0.3280 | 0.9120 | -0.0453 | 0.6265 | 0.9101 |
| Visit 4 | Renal Function Test - Creatinine -- mg/dL | 70, 0.8 (0.29, 6.21) | 76, 0.8 (0.09, 9.86) | -0.09 | 0.3325 | 0.9120 | -0.0621 | 0.5295 | 0.9101 |
| Visit 4 | Renal Function Test - Glucose -- mg/dL | 70, 110.5 (64.0, 250.0) | 76, 119.0 (63.0, 438.0) | -0.246 | 0.3964 | 0.9248 | -0.1690 | 0.0998 | 0.9101 |
| Visit 4 | Renal Function Test - Phosphorus -- mg/dL | 40, 3.3 (1.4, 9.3) | 28, 3.3499999999999996 (2.2, 6.7) | -0.091 | 0.5870 | 0.9595 | -0.0305 | 0.8252 | 0.9235 |
| Visit 4 | Renal Function Test - Potassium -- mmol/L | 69, 4.1 (3.2, 5.5) | 76, 4.2 (3.2, 5.6) | -0.162 | 0.1766 | 0.6181 | -0.1049 | 0.2659 | 0.9101 |
| Visit 4 | Renal Function Test - Sodium -- mmol/L | 69, 138.0 (132.0, 145.0) | 76, 138.0 (123.0, 145.0) | 0.271 | 0.1681 | 0.6181 | 0.1581 | 0.1086 | 0.9101 |
| Visit 4 | Renal Function Test - eGFR If African American -- mL/min/1.73 | 15, 89.0 (21.0, 189.0) | 22, 93.6 (5.0, 215.1) | -0.068 | 0.8406 | 0.9595 | 0.0284 | 0.9107 | 0.9383 |
| Visit 4 | Renal Function Test - eGFR If NonAfrican American -- mL/min/1.73 | 22, 90.0 (12.0, 146.9) | 17, 89.0 (46.0, 147.0) | -0.109 | 0.9321 | 0.9595 | -0.1075 | 0.5895 | 0.9101 |
| Visit 4 | Troponin -- ng/mL | 23, 0.01 (0.004, 0.149) | 13, 0.02 (0.003, 0.164) | -0.297 | 0.3387 | 0.9120 |  |  |  |
| Visit 5 | Albumin -- g/dL | 79, 4.2 (1.9, 5.1) | 103, 4.2 (2.4, 5.2) | 0.116 | 0.3269 | 0.7628 | 0.0800 | 0.3490 | 0.9034 |
| Visit 5 | C-reactive protein (CRP) -- mg/L | 53, 5.4 (0.2, 61.0) | 63, 5.0 (1.0, 56.2) | 0.157 | 0.4857 | 0.8499 | 0.0688 | 0.5211 | 0.9584 |
| Visit 5 | CBC - Absolute Lymphocyte Count -- 10^3/uL | 73, 1.82 (0.26, 6.2) | 99, 2.1 (0.3, 32.6) | -0.18 | 0.1247 | 0.5457 | -0.1573 | 0.4388 | 0.9034 |
| Visit 5 | CBC - Absolute Neutrophil Count -- 10^3/uL | 73, 3.94 (0.93, 9.2) | 100, 3.9 (1.2, 9.79) | -0.08 | 0.7388 | 0.9416 | -0.0337 | 0.6985 | 0.9584 |
| Visit 5 | CBC - Hematocrit (HCT) -- % | 79, 41.0 (19.8, 51.5) | 105, 40.3 (24.1, 47.9) | 0.249 | 0.0755 | 0.5117 | 0.1259 | 0.1576 | 0.6893 |
| Visit 5 | CBC - Hemoglobin (HGB) -- g/dL | 79, 13.4 (6.9, 17.1) | 105, 13.1 (8.4, 16.7) | 0.269 | 0.0695 | 0.5117 | 0.1460 | 0.0975 | 0.6823 |
| Visit 5 | CBC - Platelet Count (PLT) -- 10^3/uL | 79, 274.0 (22.0, 518.0) | 104, 248.5 (98.0, 526.0) | 0.011 | 0.7215 | 0.9416 | 0.0706 | 0.4228 | 0.9034 |
| Visit 5 | CBC - Red Blood Cell Count (RBC) -- 10^6/uL | 78, 4.66 (2.03, 5.69) | 105, 4.59 (2.41, 5.69) | 0.087 | 0.3110 | 0.7628 | 0.0418 | 0.6278 | 0.9584 |
| Visit 5 | CBC - White Blood Cell Count (WBC) -- 10^3/uL | 78, 6.58 (2.5, 13.2) | 105, 6.8 (2.3, 12.7) | -0.041 | 0.7001 | 0.9416 | 0.0117 | 0.8916 | 0.9584 |
| Visit 5 | Creatinine Kinase -- U/L | 75, 92.0 (21.0, 667.0) | 97, 102.0 (20.0, 589.0) | -0.041 | 0.4709 | 0.8499 | -0.0396 | 0.6472 | 0.9584 |
| Visit 5 | D-Dimer -- ug/mL | 63, 0.4 (0.11, 2.47) | 80, 0.39 (0.1, 27.0) | -0.171 | 0.8071 | 0.9416 | -0.4168 | 0.2963 | 0.9034 |
| Visit 5 | Ferritin -- ng/mL | 76, 69.0 (2.0, 3934.0) | 97, 86.0 (6.0, 7204.0) | -0.072 | 0.7854 | 0.9416 | -0.0048 | 0.9584 | 0.9584 |
| Visit 5 | Lactate dehydrogenase -- U/L | 77, 209.0 (113.0, 573.0) | 97, 197.0 (115.0, 456.0) | 0.27 | 0.0595 | 0.5117 | 0.1495 | 0.0925 | 0.6823 |
| Visit 5 | Liver Function Test - ALT (SGPT) -- U/L | 78, 21.5 (9.0, 75.0) | 101, 19.0 (7.0, 282.0) | -0.09 | 0.1471 | 0.5719 | -0.0194 | 0.8285 | 0.9584 |
| Visit 5 | Liver Function Test - AST (SGOT) -- U/L | 79, 21.0 (11.0, 51.0) | 102, 18.5 (9.0, 125.0) | -0.032 | 0.1015 | 0.5117 | 0.0170 | 0.8412 | 0.9584 |
| Visit 5 | Liver Function Test - Alkaline Phosphatase -- U/L | 79, 73.0 (40.0, 210.0) | 102, 82.5 (35.0, 217.0) | -0.269 | 0.0521 | 0.5117 | -0.2074 | 0.0350 | 0.6823 |
| Visit 5 | Liver Function Test - Bilirubin, Direct -- mg/dL | 52, 0.14 (0.1, 0.8) | 66, 0.19 (0.0, 1.2) | -0.418 | 0.0324 | 0.5117 | -0.3139 | 0.0489 | 0.6823 |
| Visit 5 | Liver Function Test - Bilirubin, Total -- mg/dL | 78, 0.4 (0.2, 5.7) | 102, 0.5 (0.2, 9.5) | -0.187 | 0.2715 | 0.7628 | -0.1068 | 0.4147 | 0.9034 |
| Visit 5 | Liver Function Test - Protein, Total -- g/dL | 79, 7.3 (5.9, 8.4) | 102, 7.3 (6.1, 9.0) | -0.041 | 0.8387 | 0.9469 | 0.0101 | 0.9065 | 0.9584 |
| Visit 5 | Partial Thromboplastin Time (PTT) -- seconds | 72, 28.85 (21.9, 93.0) | 89, 28.9 (23.0, 40.0) | 0.183 | 0.7634 | 0.9416 | 0.0803 | 0.4241 | 0.9034 |
| Visit 5 | Procalcitonin -- ng/mL | 62, 0.04 (0.01, 0.61) | 87, 0.04 (0.01, 0.98) | -0.026 | 0.5882 | 0.9358 | -0.0266 | 0.7807 | 0.9584 |
| Visit 5 | Prothrombin Time (PT) -- seconds | 73, 12.3 (9.8, 27.1) | 91, 12.4 (9.9, 21.3) | 0.064 | 0.6633 | 0.9416 | 0.0081 | 0.9274 | 0.9584 |
| Visit 5 | Renal Function Test - BUN -- mg/dL | 80, 13.0 (5.0, 85.0) | 106, 14.0 (4.0, 116.0) | -0.06 | 0.3494 | 0.7642 | -0.0941 | 0.3198 | 0.9034 |
| Visit 5 | Renal Function Test - BUN/Creatinine Ratio | 77, 15.1 (6.1, 30.0) | 105, 16.0 (2.8, 35.0) | 0.028 | 0.9274 | 0.9538 | -0.0402 | 0.6425 | 0.9584 |
| Visit 5 | Renal Function Test - Calcium -- mg/dL | 80, 9.4 (8.0, 10.9) | 106, 9.5 (6.6, 10.8) | 0.118 | 0.9538 | 0.9538 | 0.0473 | 0.5762 | 0.9584 |
| Visit 5 | Renal Function Test - Carbon Dioxide, Total -- mmol/L | 80, 25.0 (18.0, 32.0) | 106, 25.0 (17.0, 33.0) | -0.207 | 0.1023 | 0.5117 | -0.1486 | 0.0828 | 0.6823 |
| Visit 5 | Renal Function Test - Chloride -- mmol/L | 80, 104.0 (93.0, 111.0) | 106, 104.0 (92.0, 118.0) | -0.024 | 0.8921 | 0.9538 | -0.0066 | 0.9370 | 0.9584 |
| Visit 5 | Renal Function Test - Creatinine -- mg/dL | 80, 0.825 (0.38, 5.89) | 106, 0.87 (0.39, 10.71) | -0.198 | 0.2501 | 0.7628 | -0.1541 | 0.1519 | 0.6893 |
| Visit 5 | Renal Function Test - Glucose -- mg/dL | 80, 96.5 (38.0, 793.0) | 106, 95.0 (62.0, 476.0) | -0.096 | 0.5440 | 0.9067 | -0.0744 | 0.4219 | 0.9034 |
| Visit 5 | Renal Function Test - Phosphorus -- mg/dL | 56, 3.4 (1.9, 4.7) | 74, 3.3 (2.0, 8.6) | 0.02 | 0.3179 | 0.7628 | 0.0373 | 0.7099 | 0.9584 |
| Visit 5 | Renal Function Test - Potassium -- mmol/L | 80, 4.0 (3.1, 5.1) | 106, 4.1 (3.1, 6.2) | -0.157 | 0.4187 | 0.8142 | -0.1081 | 0.2180 | 0.8477 |
| Visit 5 | Renal Function Test - Sodium -- mmol/L | 80, 140.0 (134.0, 146.0) | 106, 140.0 (133.0, 145.0) | 0.016 | 0.9039 | 0.9538 | -0.0257 | 0.7608 | 0.9584 |
| Visit 5 | Renal Function Test - eGFR If African American -- mL/min/1.73 | 36, 101.5 (8.0, 173.0) | 56, 100.5 (7.0, 160.0) | 0.117 | 0.7611 | 0.9416 | 0.2279 | 0.1338 | 0.6893 |
| Visit 5 | Renal Function Test - eGFR If NonAfrican American -- mL/min/1.73 | 35, 87.0 (23.0, 127.0) | 49, 95.0 (6.0, 166.0) | -0.132 | 0.3138 | 0.7628 | 0.0399 | 0.7802 | 0.9584 |
| Visit 5 | Troponin -- ng/mL | 34, 0.01 (0.008, 0.194) | 50, 0.01 (0.01, 0.142) | -0.026 | 0.3981 | 0.8142 | -0.0290 | 0.8185 | 0.9584 |

**Supplementary Table 5:** Results from multivariable modeling to search for important risk factors among clinical features that were found significant in univariate analysis. Model 1 is the final selected model from the modeling process that included renal carbon dioxide. Model 2 is the final selected model from the modeling process that excluded renal carbon dioxide, as sensitivity analysis.

| **Model** | **N** | **Selected risk factors** | **Estimate (StdErr) of regression coefficient** | **P-value** |
| --- | --- | --- | --- | --- |
| Model 1 | 122 | Whether dexamethasone was administered | 2.60 (0.72) | <0.001 |
|  |  | Hospital duration in days | 0.16 (0.05) | 0.0019 |
|  |  | WHO score | -1.97 (0.63) | 0.0018 |
|  |  | CBC - Absolute Lymphocyte Count (Visit 1) | -0.84 (0.43) | 0.048 |
|  |  | Renal Function Test - Carbon Dioxide, Total (Visit 4) | 2.50 (1.44) | 0.081 |
|  |  | Whether BMI >= 30 kg/m2 | 0.65 (0.45) | 0.145 |
| Model 2 | 285 | Whether dexamethasone was administered | 1.30 (0.39) | <0.001 |
|  |  | Hospital duration in days | 0.15 (0.04) | <0.001 |
|  |  | WHO score | -0.50 (0.28) | 0.075 |
|  |  | CBC - Absolute Lymphocyte Count (Visit 1) | -0.52 (0.24) | 0.032 |
|  |  | Whether someone uses tobacco | 0.76 (0.31) | 0.013 |
|  |  | Whether someone has GERD | 1.36 (0.63) | 0.031 |
|  |  | Race - Asian vs. non-Hispanic White | -1.19 (0.78) | 0.128 |
|  |  | Race - Black or African American vs. non-Hispanic White | -0.93 (0.39) | 0.016 |
|  |  | Race - Other vs. non-Hispanic White | -1.29 (0.38) | <0.001 |
|  |  | Race - Unknown vs. non-Hispanic White | 0.93 (1.22) | 0.446 |

**Supplementary Table 6:** Demographics and comorbidities of symptom clusters. Statistical significance between each pair of clusters is labeled using * to indicate statistical significant difference between Cluster 1 and 2, $ for Cluster 1 and 3, and # for Cluster 2 and 3.

|  | **Cluster 1**  **(N=91)** | **Cluster 2**  **(N=23)** | **Cluster 3**  **(N=38)** |
| --- | --- | --- | --- |
| Demographics |  |  |  |
| Age, n, mean (SD) | 91, 47.5 (17.1) | 23, 50.8 (11.8)# | 38, 42.7 (14.5) |
| BMI group: >=30 kg/m2, n (%) | 40 (44.0) | 13 (56.5) | 20 (52.6) |
| Sex: Female, n (%) | 54 (59.3) | 15 (65.2) | 27 (71.1) |
| Race, n (%) |  |  |  |
| American Indian or Alaska Native | 1 (1.1) | 0 | 0 |
| Asian | 7 (7.7) | 1 (4.3) | 1 (2.6) |
| Black or African American | 23 (25.3) | 3 (13.0) | 16 (42.1) |
| White | 43 (47.3) | 13 (56.5) | 18 (47.4) |
| Other | 7 (7.7) | 2 (8.7) | 3 (7.9) |
| Unknown | 10 (11.0) | 4 (17.4) | 0 |
| Ethnicity, n (%) |  |  |  |
| Hispanic | 31 (34.1) | 7 (30.4) | 5 (13.2)$ |
| Non-Hispanic | 57 (62.6) | 14 (60.9) | 33 (86.8) |
| Unknown | 3 (3.3) | 2 (8.7) | 0 |
| Race - ethnic group, n (%) |  |  |  |
| Asian | 7 (8.0) | 1 (4.8) | 1 (2.6)$# |
| Black or African American | 23 (26.1) | 3 (14.3) | 16 (42.1) |
| Non-Hispanic White | 21 (23.9) | 9 (42.9) | 15 (39.5) |
| Other | 37 (42.0) | 8 (38.1) | 6 (15.8) |
| Unknown | 3 (3.3) | 2 (8.7) | 0 |
| Tobacco use: Yes, n (%) | 23 (25.3) | 5 (21.7) | 11 (28.9) |
| Comorbidities, n (%) |  |  |  |
| Anemia | 7 (7.7) | 1 (4.3) | 2 (5.3) |
| Asthma | 7 (7.7) | 2 (8.7) | 7 (18.4) |
| Atrial fibrillation | 2 (2.2) | 1 (4.3) | 2 (5.3) |
| Chronic kidney disease | 4 (4.4) | 1 (4.3) | 0 (0.0) |
| Coronary artery disease | 4 (4.4) | 2 (8.7) | 2 (5.3) |
| Gastroesophageal reflux disease | 6 (6.6) | 4 (17.4) | 3 (7.9) |
| Hyperlipidaemia | 12 (13.2) | 4 (17.4) | 2 (5.3) |
| Hypertension | 37 (40.7) | 11 (47.8) | 12 (31.6) |
| Type 2 diabetes mellitus | 19 (20.9) | 4 (17.4) | 3 (7.9) |
| Concomitant medication, n (%) |  |  |  |
| Acetaminophen | 58 (63.7) | 15 (65.2) | 20 (52.6) |
| Azithromycin | 14 (15.4) | 3 (13.0) | 3 (7.9) |
| Ceftriaxone | 10 (11.0) | 4 (17.4) | 1 (2.6) |
| Dexamethasone | 41 (45.1) | 12 (52.2) | 14 (36.8) |
| Enoxaparin | 38 (41.8) | 12 (52.2) | 12 (31.6) |
| Furosemide | 9 (9.9) | 3 (13.0) | 1 (2.6) |
| Heparin | 5 (5.5) | 0 (0.0) | 3 (7.9) |
| Ibuprofen | 11 (12.1) | 4 (17.4) | 3 (7.9) |
| Remdesivir | 34 (37.4) | 9 (39.1) | 10 (26.3) |

**Supplementary Table 7:** Regression analysis results of change in symptom count from enrollment (Visit 1) to the 3-month follow-up visit (Visit 5) among 3 symptom clusters, adjusting for Days from COVID start to Visit 5.

| **N** | **Parameter** | **Estimate (StdErr) of the Parameter** | **P-value** |
| --- | --- | --- | --- |
| 152 | Intercept | 6.58 (0.99) | <0.001 |
|  | Days from COVID start to Visit 5 | -0.01 (0.01) | 0.042 |
|  | Cluster 1 vs. Cluster 3 | -9.53 (0.89) | <0.001 |
|  | Cluster 2 vs. Cluster 3 | -7.60 (1.18) | <0.001 |

**Supplementary Table 8:** Clinical lab comparisons of symptom clusters at enrollment, discharge, and follow-up 3 months after hospital presentation (Visit 5). Statistical significance between each pair of clusters is labeled using * to indicate statistical significant difference between Cluster 1 and 2, $ for Cluster 1 and 3, and # for Cluster 2 and 3.

| **Visit** | **Clinical Lab** | **Cluster 1**  **(N=91)**  **n, median (min, max)** | **Cluster 2**  **(N=23)**  **n, median (min, max)** | **Cluster 3**  **(N=38)**  **n, median (min, max)** |
| --- | --- | --- | --- | --- |
| Visit 1 | Albumin -- g/dL | 76, 3.6 (2.0, 4.9) | 22, 3.75 (2.6, 5.0) | 36, 3.85 (2.9, 4.6) |
| Visit 1 | C-reactive protein (CRP) -- mg/L | 66, 32.05 (2.0, 202.0) | 16, 20.1 (3.0, 800.0) | 28, 21.25 (3.0, 127.3) |
| Visit 1 | CBC - Absolute Lymphocyte Count -- 10^3/uL | 69, 1.09 (0.42, 3.63) | 20, 0.98 (0.3, 2.3) | 35, 1.22 (0.18, 4.51) |
| Visit 1 | CBC - Absolute Neutrophil Count -- 10^3/uL | 61, 3.6 (0.8, 78.5) | 18, 3.53 (1.5, 10.6) | 35, 3.21 (0.88, 11.4) |
| Visit 1 | CBC - Hematocrit (HCT) -- % | 87, 39.1 (24.5, 58.6) | 22, 40.0 (29.7, 47.8) | 38, 39.85 (26.8, 48.9) |
| Visit 1 | CBC - Hemoglobin (HGB) -- g/dL | 87, 13.0 (8.6, 18.7) | 22, 13.7 (9.2, 16.6) | 38, 13.1 (8.9, 16.5) |
| Visit 1 | CBC - Platelet Count (PLT) -- 10^3/uL | 86, 228.5 (98.0, 546.0) | 22, 253.0 (133.0, 432.0) | 38, 256.0 (78.0, 493.0) |
| Visit 1 | CBC - Red Blood Cell Count (RBC) -- 10^6/uL | 87, 4.52 (2.86, 6.69) | 22, 4.685 (4.04, 5.56) | 38, 4.655 (2.36, 6.11) |
| Visit 1 | CBC - White Blood Cell Count (WBC) -- 10^3/uL | 87, 5.7 (1.7, 18.86) | 22, 5.22 (2.9, 12.6) | 38, 4.9 (2.1, 16.09) |
| Visit 1 | Creatinine Kinase -- U/L | 45, 62.0 (17.0, 1465.0) | 11, 100.0 (46.0, 332.0) | 32, 73.0 (16.0, 1094.0) |
| Visit 1 | D-Dimer -- ug/mL | 64, 0.53 (0.1, 6.934) | 15, 0.6 (0.16, 2.31) | 30, 0.4 (0.19, 10.26) |
| Visit 1 | Ferritin -- ng/mL | 59, 282.7 (24.0, 13152.0) | 14, 265.0 (14.0, 3458.0) | 33, 153.0 (7.0, 5823.0)$ |
| Visit 1 | Lactate dehydrogenase -- U/L | 54, 256.5 (114.0, 910.0) | 13, 252.0 (147.0, 968.0) | 34, 220.0 (127.0, 602.0) |
| Visit 1 | Liver Function Test - ALT (SGPT) -- U/L | 75, 26.0 (6.0, 332.0) | 22, 22.5 (9.0, 434.0) | 36, 21.5 (8.0, 85.0) |
| Visit 1 | Liver Function Test - AST (SGOT) -- U/L | 75, 27.0 (11.0, 201.0) | 22, 30.5 (14.0, 572.0) | 37, 22.0 (14.0, 81.0) |
| Visit 1 | Liver Function Test - Alkaline Phosphatase -- U/L | 76, 69.0 (28.0, 165.0) | 22, 71.0 (39.0, 217.0) | 37, 66.0 (44.0, 147.0) |
| Visit 1 | Liver Function Test - Bilirubin, Direct -- mg/dL | 48, 0.2 (0.0, 1.0) | 14, 0.2 (0.1, 0.4) | 26, 0.185 (0.0, 0.75) |
| Visit 1 | Liver Function Test - Bilirubin, Total -- mg/dL | 77, 0.4 (0.2, 8.1) | 22, 0.5 (0.2, 1.2) | 36, 0.4 (0.1, 2.8) |
| Visit 1 | Liver Function Test - Protein, Total -- g/dL | 77, 6.9 (4.6, 9.0) | 22, 6.95 (5.6, 8.2) | 37, 7.2 (6.1, 8.3) |
| Visit 1 | Partial Thromboplastin Time (PTT) -- seconds | 54, 30.25 (1.1, 168.0) | 13, 30.0 (25.9, 37.0) | 29, 27.9 (20.9, 47.0)$ |
| Visit 1 | Procalcitonin -- ng/mL | 48, 0.06 (0.0, 10.32) | 13, 0.05 (0.0, 0.21) | 28, 0.055 (0.01, 0.51) |
| Visit 1 | Prothrombin Time (PT) -- seconds | 59, 13.6 (9.8, 18.3) | 15, 12.7 (10.3, 15.8) | 30, 11.05 (10.0, 23.0)$ |
| Visit 1 | Renal Function Test - BUN -- mg/dL | 87, 13.0 (5.0, 95.0) | 22, 14.0 (5.0, 20.0) | 38, 12.5 (6.0, 32.0) |
| Visit 1 | Renal Function Test - BUN/Creatinine Ratio | 41, 16.1 (4.0, 41.0) | 10, 18.3 (6.3, 42.0) | 34, 16.4 (8.0, 27.8) |
| Visit 1 | Renal Function Test - Calcium -- mg/dL | 86, 8.6 (7.0, 10.2) | 23, 8.8 (7.7, 10.4) | 38, 8.9 (7.8, 9.7) |
| Visit 1 | Renal Function Test - Carbon Dioxide, Total -- mmol/L | 82, 23.0 (17.0, 31.0) | 22, 23.5 (18.0, 34.0) | 36, 26.0 (20.0, 29.0)$ |
| Visit 1 | Renal Function Test - Chloride -- mmol/L | 87, 104.0 (91.0, 113.0) | 23, 103.0 (97.0, 109.0) | 38, 104.0 (99.0, 108.0) |
| Visit 1 | Renal Function Test - Creatinine -- mg/dL | 87, 0.8 (0.4, 8.3) | 23, 0.8 (0.43, 1.21) | 38, 0.805 (0.49, 2.58) |
| Visit 1 | Renal Function Test - Glucose -- mg/dL | 87, 109.0 (0.6, 479.0) | 23, 120.0 (81.0, 338.0)# | 38, 104.0 (67.0, 374.0) |
| Visit 1 | Renal Function Test - Phosphorus -- mg/dL | 32, 3.35 (2.2, 5.2) | 10, 3.65 (2.7, 4.2) | 31, 3.2 (2.3, 4.8) |
| Visit 1 | Renal Function Test - Potassium -- mmol/L | 87, 4.3 (3.0, 6.6) | 23, 4.0 (3.3, 5.1)* | 38, 3.8 (3.3, 4.9)$ |
| Visit 1 | Renal Function Test - Sodium -- mmol/L | 87, 138.0 (127.0, 146.0) | 23, 138.0 (132.0, 145.0) | 38, 139.0 (133.0, 144.0) |
| Visit 1 | Renal Function Test - eGFR If African American -- mL/min/1.73 | 32, 94.5 (9.0, 141.0) | 5, 114.0 (61.0, 162.0) | 15, 110.0 (32.0, 145.0) |
| Visit 1 | Renal Function Test - eGFR If NonAfrican American -- mL/min/1.73 | 34, 90.0 (5.0, 114.0) | 14, 89.5 (49.0, 141.0) | 14, 88.0 (62.0, 120.0) |
| Visit 1 | Troponin -- ng/mL | 41, 0.01 (0.0, 1.73) | 11, 0.01 (0.007, 0.04) | 12, 0.01 (0.006, 0.04) |
| Visit 4 | Albumin -- g/dL | 19, 3.1 (2.1, 4.4) | 2, 3.1 (2.9, 3.3) | 7, 3.3 (2.9, 3.9) |
| Visit 4 | C-reactive protein (CRP) -- mg/L | 14, 23.35 (4.0, 161.2) | 3, 9.0 (2.0, 17.3) | 8, 13.05 (3.0, 82.8) |
| Visit 4 | CBC - Absolute Lymphocyte Count -- 10^3/uL | 23, 1.5 (0.3, 2.77) | 3, 1.1 (0.87, 1.4) | 11, 1.6 (0.6, 2.99) |
| Visit 4 | CBC - Absolute Neutrophil Count -- 10^3/uL | 19, 5.49 (2.4, 57.3) | 3, 6.7 (5.81, 8.5) | 11, 5.7 (2.5, 10.52) |
| Visit 4 | CBC - Hematocrit (HCT) -- % | 25, 39.6 (19.8, 58.6) | 5, 40.7 (38.2, 45.1) | 11, 39.5 (26.4, 47.6) |
| Visit 4 | CBC - Hemoglobin (HGB) -- g/dL | 25, 12.8 (7.1, 19.1) | 5, 14.4 (12.8, 15.5) | 11, 12.7 (9.3, 15.9) |
| Visit 4 | CBC - Platelet Count (PLT) -- 10^3/uL | 25, 286.0 (144.0, 538.0) | 5, 361.0 (274.0, 388.0) | 11, 355.0 (165.0, 806.0) |
| Visit 4 | CBC - Red Blood Cell Count (RBC) -- 10^6/uL | 25, 4.59 (2.16, 6.7) | 5, 4.59 (4.47, 5.06) | 11, 4.79 (2.28, 5.36) |
| Visit 4 | CBC - White Blood Cell Count (WBC) -- 10^3/uL | 25, 8.25 (4.32, 16.7) | 5, 9.3 (6.8, 10.6) | 11, 8.7 (4.5, 15.14) |
| Visit 4 | Creatinine Kinase -- U/L | 10, 70.0 (11.0, 891.0) | 2, 36.5 (23.0, 50.0) | 7, 21.0 (18.0, 147.0) |
| Visit 4 | D-Dimer -- ug/mL | 13, 0.806 (0.31, 3.9) | 3, 0.37 (0.19, 0.6) | 9, 0.6 (0.215, 1.6) |
| Visit 4 | Ferritin -- ng/mL | 13, 687.9 (41.7, 5094.0) | 3, 857.3 (103.13, 1455.0) | 10, 205.5 (68.0, 1724.0) |
| Visit 4 | Lactate dehydrogenase -- U/L | 11, 298.0 (164.0, 644.0) | 2, 281.5 (244.0, 319.0) | 11, 302.0 (208.0, 444.0) |
| Visit 4 | Liver Function Test - ALT (SGPT) -- U/L | 19, 51.0 (17.0, 158.0) | 3, 95.0 (28.0, 161.0) | 7, 52.0 (18.0, 257.0) |
| Visit 4 | Liver Function Test - AST (SGOT) -- U/L | 19, 34.0 (17.0, 101.0) | 3, 57.0 (17.0, 83.0) | 8, 36.5 (14.0, 63.0) |
| Visit 4 | Liver Function Test - Alkaline Phosphatase -- U/L | 19, 77.0 (26.0, 212.0) | 3, 51.0 (43.0, 62.0) | 8, 76.5 (58.0, 191.0) |
| Visit 4 | Liver Function Test - Bilirubin, Direct -- mg/dL | 13, 0.2 (0.1, 0.8) | 3, 0.2 (0.1, 0.2) | 6, 0.33 (0.1, 0.4) |
| Visit 4 | Liver Function Test - Bilirubin, Total -- mg/dL | 20, 0.6 (0.2, 5.5) | 3, 0.4 (0.4, 0.6) | 8, 0.8 (0.3, 1.3) |
| Visit 4 | Liver Function Test - Protein, Total -- g/dL | 20, 6.35 (5.4, 7.7) | 3, 6.0 (5.4, 6.5) | 8, 6.9 (5.7, 8.1) |
| Visit 4 | Partial Thromboplastin Time (PTT) -- seconds | 7, 34.0 (26.9, 40.6) | 2, 29.35 (29.0, 29.7) | 4, 26.4 (25.0, 27.7) |
| Visit 4 | Procalcitonin -- ng/mL | 5, 0.33 (0.05, 1.11) | 2, 0.04 (0.03, 0.05) | 5, 0.06 (0.02, 0.12) |
| Visit 4 | Prothrombin Time (PT) -- seconds | 10, 14.15 (10.9, 16.7) | 2, 14.4 (13.3, 15.5) | 8, 11.6 (10.4, 16.5) |
| Visit 4 | Renal Function Test - BUN -- mg/dL | 26, 21.0 (3.0, 65.0) | 5, 19.0 (14.0, 22.0) | 11, 17.0 (10.0, 27.0) |
| Visit 4 | Renal Function Test - BUN/Creatinine Ratio | 12, 23.05 (4.5, 58.0) | 2, 38.6 (22.2, 55.0) | 11, 23.3 (12.6, 39.1) |
| Visit 4 | Renal Function Test - Calcium -- mg/dL | 26, 8.3 (7.6, 9.1) | 5, 8.8 (8.0, 10.0) | 11, 8.9 (7.9, 11.0)$ |
| Visit 4 | Renal Function Test - Carbon Dioxide, Total -- mmol/L | 26, 24.0 (17.0, 30.0) | 5, 21.0 (8.7, 31.0) | 11, 26.0 (23.0, 34.0)$ |
| Visit 4 | Renal Function Test - Chloride -- mmol/L | 26, 104.0 (88.0, 110.0) | 5, 104.0 (99.0, 107.0) | 11, 103.0 (93.0, 105.0) |
| Visit 4 | Renal Function Test - Creatinine -- mg/dL | 26, 0.79 (0.35, 8.23) | 5, 0.63 (0.38, 1.0) | 11, 0.69 (0.43, 1.47) |
| Visit 4 | Renal Function Test - Glucose -- mg/dL | 26, 113.5 (83.0, 247.0) | 6, 122.5 (99.0, 165.0) | 11, 121.0 (78.0, 158.0) |
| Visit 4 | Renal Function Test - Phosphorus -- mg/dL | 7, 3.7 (2.6, 4.6) | 1, 3.5 (3.5, 3.5) | 9, 3.3 (2.1, 9.3) |
| Visit 4 | Renal Function Test - Potassium -- mmol/L | 26, 4.2 (3.6, 5.6) | 5, 4.2 (3.8, 4.7) | 11, 4.0 (3.2, 4.9) |
| Visit 4 | Renal Function Test - Sodium -- mmol/L | 26, 137.5 (123.0, 145.0) | 5, 135.0 (134.0, 140.0) | 11, 139.0 (135.0, 140.0) |
| Visit 4 | Renal Function Test - eGFR If African American -- mL/min/1.73 | 8, 78.0 (6.0, 184.0) | 1, 169.0 (169.0, 169.0) | 7, 113.0 (51.0, 152.0) |
| Visit 4 | Renal Function Test - eGFR If NonAfrican American -- mL/min/1.73 | 6, 80.45 (46.0, 90.0) | 4, 103.0 (77.0, 147.0) | 3, 102.0 (87.0, 118.0) |
| Visit 4 | Troponin -- ng/mL | 6, 0.01 (0.003, 0.164) | 1, 0.03 (0.03, 0.03) | 3, 0.01 (0.009, 0.149) |
| Visit 5 | Albumin -- g/dL | 41, 4.3 (3.1, 5.2) | 14, 4.2 (3.6, 5.1) | 32, 4.3 (3.6, 5.2) |
| Visit 5 | C-reactive protein (CRP) -- mg/L | 33, 5.0 (1.4, 56.2) | 12, 5.45 (1.0, 16.0) | 16, 5.6 (0.2, 40.0) |
| Visit 5 | CBC - Absolute Lymphocyte Count -- 10^3/uL | 40, 2.0 (0.4, 32.6) | 14, 1.995 (0.26, 3.8) | 33, 1.76 (0.3, 3.4) |
| Visit 5 | CBC - Absolute Neutrophil Count -- 10^3/uL | 40, 4.195 (1.4, 9.73) | 14, 4.6 (1.44, 6.6) | 33, 3.58 (1.7, 9.79) |
| Visit 5 | CBC - Hematocrit (HCT) -- % | 41, 40.5 (25.6, 47.9) | 14, 41.45 (32.9, 47.1) | 33, 40.7 (22.6, 47.1) |
| Visit 5 | CBC - Hemoglobin (HGB) -- g/dL | 41, 13.4 (8.5, 17.1) | 14, 13.4 (10.3, 16.2) | 33, 13.2 (7.9, 16.1) |
| Visit 5 | CBC - Platelet Count (PLT) -- 10^3/uL | 41, 278.0 (135.0, 430.0) | 14, 270.5 (152.0, 364.0) | 33, 263.0 (104.0, 451.0) |
| Visit 5 | CBC - Red Blood Cell Count (RBC) -- 10^6/uL | 41, 4.59 (2.58, 5.41) | 14, 4.74 (3.76, 5.37) | 33, 4.66 (2.41, 5.64) |
| Visit 5 | CBC - White Blood Cell Count (WBC) -- 10^3/uL | 41, 7.09 (2.3, 12.2) | 14, 7.0 (3.5, 10.4) | 33, 6.48 (3.7, 12.7) |
| Visit 5 | Creatinine Kinase -- U/L | 39, 84.0 (32.0, 667.0) | 14, 127.5 (51.0, 571.0) | 31, 99.0 (48.0, 241.0) |
| Visit 5 | D-Dimer -- ug/mL | 32, 0.355 (0.135, 2.24) | 11, 0.3 (0.19, 1.02) | 21, 0.4 (0.135, 1.87) |
| Visit 5 | Ferritin -- ng/mL | 39, 60.0 (6.0, 7204.0) | 14, 66.5 (11.0, 223.0) | 31, 39.0 (2.0, 3687.0) |
| Visit 5 | Lactate dehydrogenase -- U/L | 39, 204.0 (139.0, 321.0) | 13, 200.0 (136.0, 262.0) | 32, 184.5 (115.0, 559.0) |
| Visit 5 | Liver Function Test - ALT (SGPT) -- U/L | 39, 17.0 (7.0, 108.0) | 14, 20.5 (13.0, 56.0) | 31, 18.0 (10.0, 60.0) |
| Visit 5 | Liver Function Test - AST (SGOT) -- U/L | 40, 18.5 (9.0, 47.0) | 14, 20.5 (12.0, 47.0) | 32, 19.5 (14.0, 38.0) |
| Visit 5 | Liver Function Test - Alkaline Phosphatase -- U/L | 40, 82.5 (35.0, 145.0) | 14, 71.0 (47.0, 121.0) | 32, 71.5 (45.0, 125.0) |
| Visit 5 | Liver Function Test - Bilirubin, Direct -- mg/dL | 21, 0.16 (0.0, 1.2) | 7, 0.1 (0.1, 0.38) | 25, 0.2 (0.1, 0.71) |
| Visit 5 | Liver Function Test - Bilirubin, Total -- mg/dL | 40, 0.45 (0.2, 9.5) | 14, 0.45 (0.2, 1.0) | 31, 0.5 (0.2, 2.6) |
| Visit 5 | Liver Function Test - Protein, Total -- g/dL | 40, 7.35 (6.1, 9.0) | 14, 7.15 (6.2, 8.0)# | 32, 7.45 (6.2, 8.5) |
| Visit 5 | Partial Thromboplastin Time (PTT) -- seconds | 35, 29.2 (23.0, 40.0) | 13, 29.6 (24.5, 50.7) | 30, 28.1 (21.9, 93.0) |
| Visit 5 | Procalcitonin -- ng/mL | 35, 0.06 (0.01, 0.21) | 11, 0.06 (0.01, 0.06) | 30, 0.03 (0.01, 0.61)$ |
| Visit 5 | Prothrombin Time (PT) -- seconds | 35, 13.0 (9.9, 18.0) | 13, 12.8 (10.2, 19.1) | 31, 11.4 (9.8, 24.1) |
| Visit 5 | Renal Function Test - BUN -- mg/dL | 41, 13.0 (5.0, 116.0) | 14, 12.5 (10.0, 26.0) | 32, 12.0 (4.0, 19.0) |
| Visit 5 | Renal Function Test - BUN/Creatinine Ratio | 41, 15.9 (3.6, 32.2) | 14, 17.0 (9.0, 23.0) | 32, 14.0 (4.9, 35.0) |
| Visit 5 | Renal Function Test - Calcium -- mg/dL | 41, 9.4 (7.1, 10.4) | 14, 9.65 (8.8, 10.9) | 32, 9.5 (8.4, 10.4) |
| Visit 5 | Renal Function Test - Carbon Dioxide, Total -- mmol/L | 41, 25.0 (19.0, 32.0) | 14, 25.0 (18.0, 31.0) | 32, 26.0 (20.0, 30.0) |
| Visit 5 | Renal Function Test - Chloride -- mmol/L | 41, 105.0 (97.0, 110.0) | 14, 104.0 (100.0, 111.0) | 32, 104.0 (98.0, 109.0) |
| Visit 5 | Renal Function Test - Creatinine -- mg/dL | 41, 0.81 (0.52, 5.58) | 14, 0.855 (0.54, 1.52) | 32, 0.795 (0.51, 1.29) |
| Visit 5 | Renal Function Test - Glucose -- mg/dL | 41, 92.0 (54.0, 356.0) | 14, 115.0 (64.0, 192.0)# | 32, 88.5 (62.0, 302.0) |
| Visit 5 | Renal Function Test - Phosphorus -- mg/dL | 23, 3.1 (2.1, 8.6) | 7, 3.5 (2.7, 3.7) | 27, 3.3 (1.9, 4.6) |
| Visit 5 | Renal Function Test - Potassium -- mmol/L | 41, 4.2 (3.5, 6.2) | 14, 4.1 (3.5, 4.6) | 32, 3.9 (3.1, 4.5)$ |
| Visit 5 | Renal Function Test - Sodium -- mmol/L | 41, 140.0 (133.0, 145.0) | 14, 140.0 (137.0, 146.0) | 32, 141.0 (137.0, 144.0) |
| Visit 5 | Renal Function Test - eGFR If African American -- mL/min/1.73 | 29, 108.0 (10.0, 160.0) | 10, 82.0 (46.0, 150.0) | 13, 102.0 (61.0, 158.0) |
| Visit 5 | Renal Function Test - eGFR If NonAfrican American -- mL/min/1.73 | 20, 99.5 (21.0, 138.0) | 10, 76.0 (49.0, 130.0) | 10, 95.5 (63.0, 121.0) |
| Visit 5 | Troponin -- ng/mL | 21, 0.01 (0.01, 0.01) | 8, 0.01 (0.01, 0.02) | 9, 0.01 (0.01, 0.194)$ |

**Supplementary Table 9:** Summary of system organ class (SOC) of symptom clusters at enrollment and at the 3-month follow-up visit (Visit 5). Statistical significance between each pair of clusters is labeled using * to indicate statistical significant difference between Cluster 1 and 2, $ for Cluster 1 and 3, and # for Cluster 2 and 3. “Other” is for symptoms that were reported as “Other” at the time when a participant filled out the outcome survey. The full list of symptoms collected in the outcome survey is shown in Supplementary Table 2.

| **System organ class (SOC)** | **Cluster 1**  **(N=91)**  **n (%)** | **Cluster 2**  **(N=23)**  **n (%)** | **Cluster 3**  **(N=38)**  **n (%)** |
| --- | --- | --- | --- |
| SOC of symptoms collected at study entry |  |  |  |
| Gastrointestinal disorders | 32 (35.2) | 11 (47.8) | 15 (39.5) |
| General disorders and administration site conditions | 75 (82.4) | 21 (91.3) | 36 (94.7) |
| Injury, poisoning and procedural complications | 1 (1.1) | 1 (4.3) | 0 |
| Metabolism and nutrition disorders | 27 (29.7) | 5 (21.7) | 17 (44.7) |
| Nervous system disorders | 57 (62.6) | 20 (87.0)* | 35 (92.1)$ |
| Psychiatric disorders | 8 (8.8) | 3 (13.0) | 7 (18.4) |
| Respiratory, thoracic and mediastinal disorders | 73 (80.2) | 20 (87.0) | 36 (94.7) |
| SOC of symptoms collected at the 3-month follow-up visit (Visit 5) |  |  |  |
| Eye disorders | 1 (1.1) | 2 (8.7) | 10 (26.3)$ |
| Gastrointestinal disorders | 4 (4.4) | 5 (21.7)*# | 23 (60.5)$ |
| General disorders and administration site conditions | 43 (47.3) | 22 (95.7)* | 38 (100.0)$ |
| Injury, poisoning and procedural complications | 0 | 0 | 3 (7.9)$ |
| Metabolism and nutrition disorders | 6 (6.6) | 0 | 16 (42.1)$ |
| Musculoskeletal and connective tissue disorders | 8 (8.8) | 5 (21.7)# | 32 (84.2)$ |
| Nervous system disorders | 10 (11.0) | 13 (56.5)*# | 36 (94.7)$ |
| Other | 3 (3.3) | 3 (13.0) | 5 (13.2) |
| Psychiatric disorders | 2 (2.2) | 11 (47.8)* | 26 (68.4)$ |
| Respiratory, thoracic and mediastinal disorders | 32 (35.2) | 17 (73.9)*# | 37 (97.4)$ |
| Skin and subcutaneous tissue disorders | 1 (1.1) | 1 (4.3) | 7 (18.4)$ |

**Supplementary Table 10:** Symptom changes between clusters at enrollment versus recovery. Each participant was categorized into one of four groups for each SOC, based on whether they had a symptom in that category at enrollment and at the 3-month follow-up visit (Visit 5).

| **SOC** | **Type of symptom change** | **Cluster 1**  **(N=91)**  **n (%)** | **Cluster 2**  **(N=23)**  **n (%)** | **Cluster 3**  **(N=38)**  **n (%)** |
| --- | --- | --- | --- | --- |
| Gastrointestinal disorders | new onset | 4 (4.4) | 3 (13.04) | 11 (28.95) |
|  | persistent symptoms | 0 | 2 (8.7) | 12 (31.58) |
|  | never experienced | 55 (60.44) | 9 (39.13) | 12 (31.58) |
|  | recovered | 32 (35.16) | 9 (39.13) | 3 (7.89) |
| General disorders and administration site conditions | new onset | 5 (5.49) | 1 (4.35) | 2 (5.26) |
|  | persistent symptoms | 38 (41.76) | 21 (91.3) | 36 (94.74) |
|  | never experienced | 11 (12.09) | 1 (4.35) | 0 |
|  | recovered | 37 (40.66) | 0 | 0 |
| Metabolism and nutrition disorders | new onset | 3 (3.3) | 0 | 8 (21.05) |
|  | persistent symptoms | 3 (3.3) | 0 | 8 (21.05) |
|  | never experienced | 61 (67.03) | 18 (78.26) | 13 (34.21) |
|  | recovered | 24 (26.37) | 5 (21.74) | 9 (23.68) |
| Nervous system disorders | new onset | 2 (2.2) | 3 (13.04) | 2 (5.26) |
|  | persistent symptoms | 8 (8.79) | 10 (43.48) | 34 (89.47) |
|  | never experienced | 32 (35.16) | 0 | 1 (2.63) |
|  | recovered | 49 (53.85) | 10 (43.48) | 1 (2.63) |
| Psychiatric disorders | new onset | 2 (2.2) | 9 (39.13) | 20 (52.63) |
|  | persistent symptoms | 0 | 2 (8.7) | 6 (15.79) |
|  | never experienced | 81 (89.01) | 11 (47.83) | 11 (28.95) |
|  | recovered | 8 (8.79) | 1 (4.35) | 1 (2.63) |
| Respiratory, thoracic and mediastinal disorders | new onset | 1 (1.1) | 2 (8.7) | 2 (5.26) |
|  | persistent symptoms | 31 (34.07) | 15 (65.22) | 35 (92.11) |
|  | never experienced | 17 (18.68) | 1 (4.35) | 0 |
|  | recovered | 42 (46.15) | 5 (21.74) | 1 (2.63) |
